# A Genetic Algorithm Approach for Compact Wave Function Representations in Spin-Adapted Bases

**DOI:** 10.1021/acs.jctc.5c01264

**Published:** 2025-11-10

**Authors:** Maru Song, Giovanni Li Manni

**Affiliations:** 28326 Max Planck Institute for Solid State Research, 70569 Stuttgart, Germany

## Abstract

The accurate treatment of many-unpaired-electron systems remains a central challenge in quantum chemistry, due to the exponential growth of the many-electron wave function with the number of correlated electrons. *Quantum Anamorphosis* addresses this challenge through physically motivated localization of molecular orbitals and site reordering, which yield unique block-diagonal Hamiltonian matrices and compact spin-adapted many-body wave functions. In this work, we introduce a genetic algorithm to identify optimal orbital/site orderings that enhance wave function compactness, thereby enabling the study of larger systems than previously possible. Crucially, we propose fitness functions based on approximate measures of the wave function compactness, which enable inexpensive genetic algorithm searches. We benchmark the strategy against one- and two-dimensional nearest-neighbor Heisenberg models, the one-dimensional next-nearest-neighbor Heisenberg model, and *selected* collinear ground and excited states of the nitrogenase P-cluster, employing intermediate CAS­(48,40) active space ab initio Hamiltonians. In our strategy, the inclusion of nonmagnetic orbitals does not affect the fitness of the orderings, which enables the treatment of the large CAS­(114,73) active space of the P-cluster without the need to search for a new optimal ordering. These results highlight the applicability and scalability of the genetic-algorithm-driven approach for systems with many unpaired electrons. The P-cluster test case is particularly relevant, as it demonstrates that wave function compression can be applied to both collinear ground and excited states, and allows the selective targeting of electronic states expressible in the given basis.

## Graphical abstract

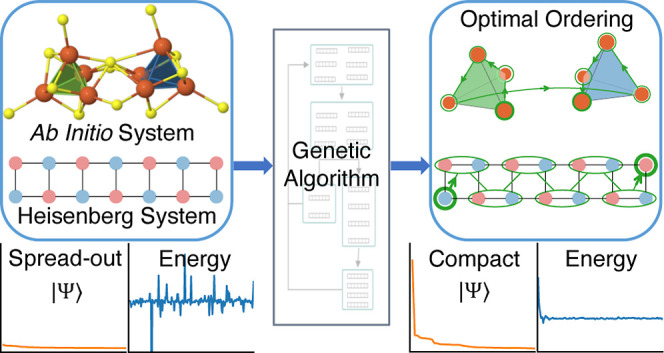

## 1 Introduction

The magnetic, catalytic, and optical properties of polynuclear transition metal (PNTM) complexes are intrinsically linked to the presence of numerous unpaired electrons in their ground, excited, and transition states. The P-cluster and the FeMo-cofactor of nitrogenase, and the Mn_12_ single-molecule magnet are prime examples. Unpaired electrons and their interactions, often mediated by bridging ligands via superexchange, also govern the properties of extended systems such as crystals. Notable examples include high-*T*_c_ superconductivity in Sr_2_CuO_3_ cuprates with corner-sharing CuO_4_ plaquettes^1^ and hole-doped infinite-layer NdNiO_2_ nickelates.^2^ In condensed matter physics, such systems are often described by effective spin Hamiltonians that capture the essential physics of interactions between magnetic centers. A prominent example is the isotropic antiferromagnetic Heisenberg Hamiltonian with nearest-neighbor (NN) interactions, commonly used to model systems dominated by exchange coupling.

The electronic structure of systems with many unpaired electrons, in practice ≳18, are generally not solvable without approximate numerical approaches. Full configuration interaction (FCI) quantum Monte Carlo (FCIQMC) has emerged as a method that can efficiently solve complex electronic structures, while explicitly correlating a number of electrons that is exceedingly large for exact many-body eigensolvers.^3–5^ In FCIQMC, the many-body electronic Hamiltonian matrix elements, $\left\langle m\left|Ĥ\right|m^{\prime }\right\rangle$ (off-diagonal terms), are stochastically sampled in the *spawning step* of the algorithm, contributing to the evolution of the many-body wave function along the imaginary-time dynamics. The efficiency of the algorithm, particularly its formulation within the *initiator approximation* (i-FCIQMC),^6–8^ is directly related to the sparsity of the Hamiltonian matrix. For large Hamiltonian matrices with a dense structure, the stochastic sampling of the $\left\langle m\left|Ĥ\right|m^{\prime }\right\rangle$ elements typically covers only a small fraction of all nonvanishing terms. As a result, the convergence of the algorithm with respect to the number of stochastic particles (*walkers*) is slow or practically compromised, leading to unstable dynamics (see Figures 8 and 9 of ref 9). On the contrary, for sparse Hamiltonian matrices, the stochastic sampling of off-diagonal terms is substantially more efficient, with the algorithm converging rapidly toward the exact solution.

The sparsity of the Hamiltonian is generally governed by the electronic system under consideration and the size and nature of the chosen model active space. Nonetheless, with those parameters fixed, the sparsity of the Hamiltonian is further affected by unitary transformations of the molecular orbitals (e.g., canonical, natural or localized orbitals). When the wave function is represented in spin-adapted bases, exchanging *orbital* or *lattice-site* indices yields an energy-invariant degree of freedom that controls the Hamiltonian’s block-diagonal structure, with important implications for the compactness of the many-body wave function. Different orbital/site orderings correspond to a *similarity transformation* of the original Hamiltonian.

Starting from 2020, as part of a multiyear project,^9–15^ we have explored ways to enhance the Hamiltonian’s block-diagonal structure by reordering the orbitals or lattice sites in the context of *graphical unitary group approach* (GUGA).^16–19^ We have named this strategy *Quantum Anamorphosis*,^20^ as it is reminiscent of the process of anamorphosis in art, where distorted sculptures reveal a recognizable structure when viewed from a specific vantage point. Similarly, in Quantum Anamorphosis, the reordering of orbitals or lattice sites transforms the Hamiltonian matrix into a (quasi-)­block-diagonal form, allowing for a more compact representation of the many-body wave function.

Formal investigations have shown that the commutation relations between the Hamiltonian operator and the cumulative partial spin operator over the first *n* magnetic sites, are responsible for the block-diagonal structure of the Hamiltonian matrix,^13,15^ namely

1 $$\left[Ĥ,{\left(\sum_{i=1}^{n}{Ŝ}_{i}\right)}^{2}\right]\to \{\begin{array}{ll} =0 & block‐diagonal\;Hamiltonian\;matrix\\ \neq 0 & dense\;Hamiltonian\;matrix \end{array}$$

When the Hamiltonian matrix is in (quasi-)­block diagonal form, the eigenvectors of the Hamiltonian are highly compact for any of the target states. Since orbital or site ordering directly affects the definition of the cumulative spin operator and its commutation with the Hamiltonian, it establishes the link between ordering, Hamiltonian’s block structure, and wave function compactness.

The permutation space generated by all possible orbital/site orderings scales factorially with the number of orbitals/sites. An exhaustive permutation-space search has been conducted on various systems with up to ten magnetic sites.^9–12,14^ For systems with more magnetic sites, an exhaustive search is impractical. While symmetry arguments reduce the size of the permutation space by approximately 1 order of magnitude,^15^ the remaining search space is still too large to be exhaustively explored.

In ref 12, a *simulated annealing* (SA) algorithm^21–24^ for the one-dimensional Heisenberg model with NN interactions was proposed. The algorithm explores the space of site permutations and basis functions, $\left\{S_{n}\right\}\times \left\{|m\rangle \right\}$ , and finds the ordering that *minimizes* the lowest diagonal matrix element

2 $$\min_{S_{n}}\;\min_{m}\;\left\langle m\left|Ĥ\right|m\right\rangle$$

During the SA optimization, the configuration state function (CSF) and site ordering are alternately optimized. For the CSF optimization part, the entire Hilbert space was explored for small systems (roughly up to 30 sites), and for larger systems, the Hilbert space was stochastically explored. As discussed in Section 3, it is precisely the minimization of the diagonal elements that prevents its extension to ab initio Hamiltonians.

The similarity between the search for an optimal ordering and the traveling salesman problem (TSP) has allowed us to explore a *genetic algorithm* (GA) to find near-optimal orderings for larger and more general Hamiltonians. The choice of the GA is motivated by the fact that this metaheuristic (a) can handle large instances (being scalable and naturally parallelizable), (b) can be tailored with custom crossover/mutation operators to diverse constrained optimizations, often the case in chemical systems (offering flexibility), and (c) can avoid local minima in virtue of its broader search space via population diversity, crossover, and mutation.

In the GA’s fitness function, the bias toward minimizing the diagonal elements of the Hamiltonian matrix has been eliminated, extending its applicability to more complex systems, including ab initio PNTM clusters. Moreover, for (unfrustrated) antiferromagnetic Heisenberg systems, where minimizing the diagonal elements of the Hamiltonian matrix is a successful strategy for finding an optimal ordering, we devised a more efficient approach, which ties the ordering to a specific CSF and eliminates the search over the CSF space, {|*m*⟩}, unlike our earlier SA minimization (eq 2).

The remainder of this paper is organized as follows: Section 2 describes our GA. The accurate and inexpensive evaluation of the fitness function is crucial for the performance of the GA; thus, in Section 3 we describe in detail the strategies that we have developed, both for spin models and ab initio systems. In Sections 4 and 5, the applicability of the GA-driven Quantum Anamorphosis is tested on spin models and ab initio systems, respectively. The isotropic antiferromagnetic Heisenberg model with NN interactions on a one-dimensional chain, *N*-leg ladders, and square lattices are the systems considered for the unfrustrated spin models. The isotropic antiferromagnetic Heisenberg model with NN and next-NN interactions (*J*_1_ – *J*_2_) on a one-dimensional chain is used to study the effect of frustration on the GA. The ab initio systems considered include a chain of eight hydrogen atoms (to relate to the corresponding spin model), an 8-site hydrogen cluster whose geometry mimics the one of the P-cluster in nitrogenase, and a more realistic nitrogenase P-cluster model, Fe­(II)_8_S_13_, in its neutral state (P^*N*^). We demonstrate that the GA can find optimal orderings for a medium-size CAS­(48,40) of the P-cluster, which consists of all metal centered valence 3d orbitals. The resulting ordering can then be transferred to a larger active space, CAS­(114,73), where nonmagnetic (3p ligand) orbitals are also included. We show that the strategy provides excellent results for all energetically low-lying collinear singlet spin states of the system, gaining exceptional control over the low-energy spectrum for this system. We conclude with a summary in Section 6. Computational details of the calculations in this work are provided in Section S1 of the Supporting Information.

## 2 Genetic Algorithm for Wave Function Compression

GA is a metaheuristic that provides near-optimal solutions to optimization problems where the search space is too large for an exhaustive search within available computational resources. Inspired by Darwin’s theory of evolution by natural selection, GA emulates the evolutionary process by evolving a population of solutions over generations.^25–27^ In this process, the solutions with high fitness scores are more likely to survive and pass their genetic information to the next generation. In each generation, the best solutions are retained to guide the search toward improved outcomes. While survival-of-the-fittest may drive convergence to local optima, maintaining population diversity helps prevent premature convergence and supports global exploration of the search space. These features set GA apart from other heuristics, such as SA or Lin–Kernighan–Helsgaun (LKH).^28–30^

GAs have been applied to various aspects of quantum chemistry methods, including the optimization of molecular geometries,^31^ reconstruction of a matrix product state (MPS) wave function into a configuration interaction (CI) wave function,^32^ and the optimization of orbital ordering for density matrix renormalization group (DMRG) calculations.^33^ It is worth noting that the orbital ordering in DMRG refers to the sequence used during the orbital sweeps when optimizing the MPS ansatz,^34–37^ whereas the orbital ordering considered in this work pertains to the cumulative orbital couplings in the Hamiltonian matrix element evaluation in GUGA. In DMRG, using an optimal ordering makes the entanglement distribution more compact and improves the convergence of the MPS ansatz with respect to the bond dimension, whereas in GUGA, an optimal ordering increases the overlap between the initial and target states. In DMRG, such an increase in overlap can be achieved, for example, by adopting entanglement-minimized orbitals.^38^ It has been shown that the best ordering for DMRG does not necessarily coincide with the best ordering within the GUGA framework for generating the most compact CI wave function.^12,14^

This work presents the first application of Genetic Algorithms to the optimization of the orbital/site ordering within the GUGA framework, aiming at maximizing the Hamiltonian’s (quasi-)­block-diagonal structure, facilitating the convergence of GUGA-FCIQMC^139^ dynamics, and providing extremely compact many-electron wave functions.

A schematic overview of all stages of the GA is provided in Figure 1, and described below. Magnetic site indices are encoded as *genes*, each site ordering as a *chromosome*, and a set of site orderings as a *population* (Figure 1a). The GA starts with a randomly generated population (0th generation). The population size, *N*_T_, is chosen as an input parameter and fixed during the simulation. For each chromosome in the population, a *fitness score* is evaluated by a fitness function. Using a fitness function that is a good proxy for the wave function compactness with low computational cost is crucial for the performance of the algorithm, and will be discussed in greater details in Section 3.

**Figure 1:**
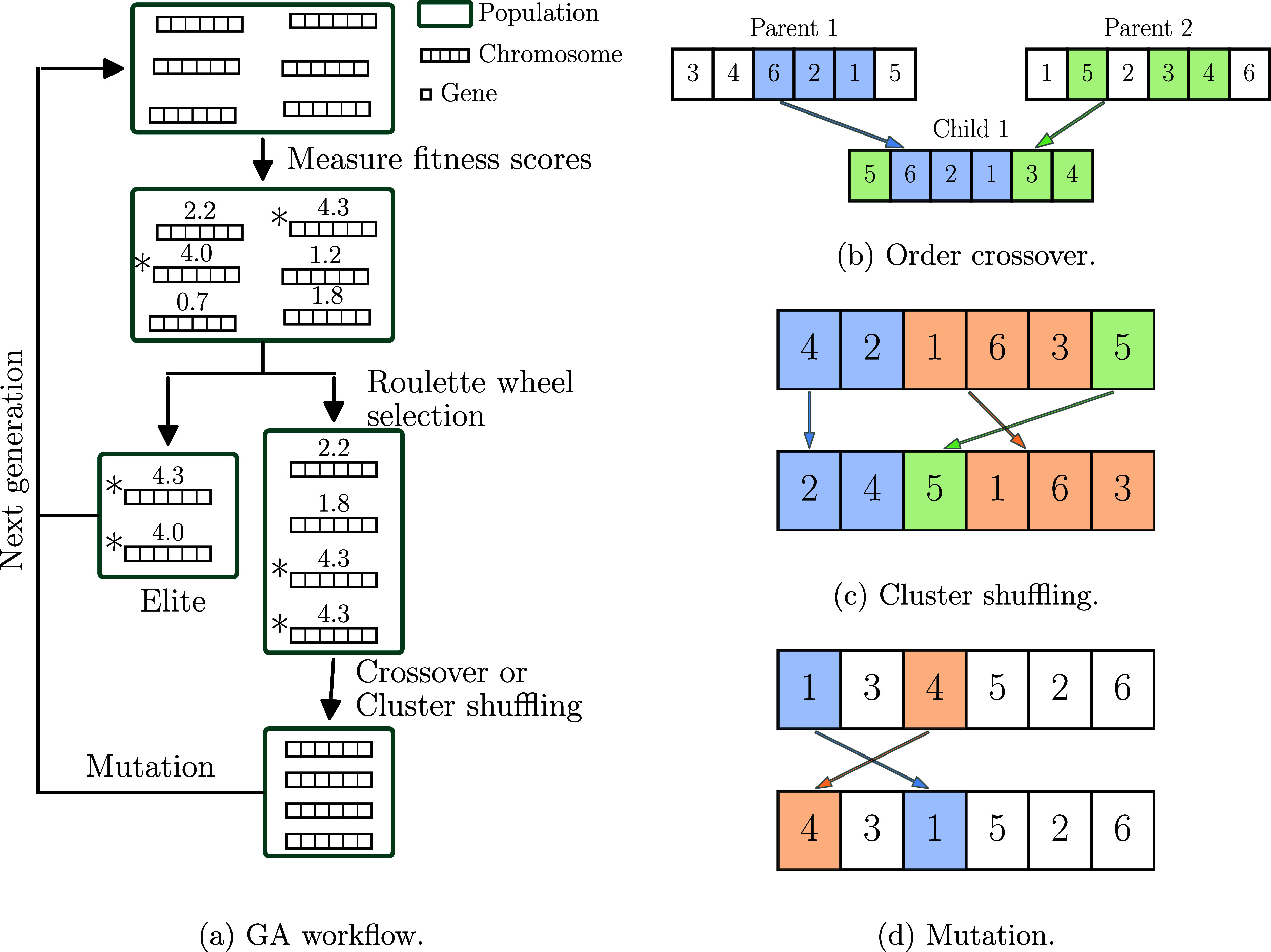
GA workflow and its main steps. (a) The fitness scores are shown above the chromosomes and elite chromosomes are marked with asterisks. (b) A random gene sequence (blue) from the first parent is inserted in the first child; the rest comes from the second parent (green). Roles switch for the second child. (c) With 10% probability, clusters are reversed (blue), then two random clusters (red, green) are swapped. (d) One or more gene pairs may swap with a preset probability.

*Elite* chromosomes—those with the highest fitness scores—are selected from the current population and directly passed to the next generation. The number of elite chromosomes, *N*_E_, is treated as an input parameter, and in the test cases, it was kept to 10% of the total population (*N*_E_ = 0.1 × *N*_T_). Based on fitness scores, (*N*_T_ – *N*_E_) chromosomes are sampled from the population (including elites) with probability proportional to their fitness score (roulette wheel selection) to form the *mating pool*. Chromosomes with high fitness scores are likely to be sampled multiple times (duplicates). For convenience, we always chose an even number of *N*_T_ and *N*_E_ in our simulations.

Next, the chromosomes in the mating pool are randomly paired (*parents*). A *crossover* is performed for each pair of parents to generate two *children*. Due to the restriction that all genes (sites) must appear only once in a chromosome, we use *order crossover*.^39^ In the order crossover (Figure 1b), a sequence of adjacent genes is taken from the first parent. The position and length of the sequence is randomly selected, and the sequence takes a random position in the child chromosome. To fill the remaining gene positions for the child, the genes of the second parent are scanned from its first position. If the gene already exists in the sequence from the first parent, then the next gene of the second parent is checked; otherwise, the gene is added to the first empty position in the child. Empty positions are filled in order. After the first child is generated, the parents change their roles.

Local minima exist in which chromosomes have gene sequences (*site clusters*) that also appear in the best chromosome, but in different positions. We have devised an additional *cluster shuffling* (CS) step that replaces the crossover step every few generations (in our tests every five generations unless otherwise specified), helping the simulation escapes local minima and guiding it toward the global optimum. In the CS step (Figure 1c), chromosomes in the mating pool are split into multiple clusters. The number of clusters and their length of each chromosome are randomly chosen between 2 and the total number of genes in the chromosome. Once the chromosome is clustered, each cluster is reversed with 10% probability, and then two randomly chosen clusters are swapped. Despite being applied in a different domain, the CS step exhibits certain resemblance to the cluster Monte Carlo (MC) algorithms, such as the Swendsen–Wang algorithm^40^ and the Wolff algorithm,^41^ where MC moves are applied to clusters of spins rather than individual spins.

Finally, child chromosomes undergo *mutation*, also intended to prevent trapping in local optima. In the mutation step (Figure 1d), each gene in the child chromosome is swapped with a randomly chosen gene in the chromosome with a probability that is specified as an input parameter. The next generation of the population arises from merging the elites and the (mutated) children.

These evolutionary steps are iterated until the population reaches a certain preset generation. The chromosome with the highest fitness score in the final generation is then selected as the optimal ordering, yielding a highly compressed wave function for subsequent electronic structure calculations.

The algorithm described in this section has been implemented as a python package,^143^ and is also included among the auxiliary tools of OpenMolcas.^144^

## 3 Tuning the Fitness Function for Different Hamiltonians

The choice of an efficient fitness function is at the core of the performance of the GA. In the context of wave function compression, the *L*_4_ norm of the wave function represents an ideal fitness function, as it directly measures the compactness of the wave function (see ref 9 for details). However, this metric is not practical, as the *L*_4_ norm can only be exactly obtained from an already optimized wave function, which is precisely our target. Below, we propose inexpensive strategies based on the diagonal elements (CSF energies) of the many-body Hamiltonian matrix over specific CSFs, exclusively evaluated over the exchange interaction part of the Hamiltonian operator.

Within Quantum Anamorphosis, wave function compression is largely related to the cumulative spin coupling of the unpaired electrons. It is thus possible to maximize compression over the van Vleck–Sherman subspace, which consists exclusively of CSFs with cumulative spin-up (*u*) and spin-down (*d*) couplings (CSFs^vvs^) out of the four possible couplings (0, *u*, *d*, and 2, where 0 and 2 represent empty and doubly occupied orbital couplings, respectively). As an example, a system with 3 orbitals and 3 electrons at *S* = 1/2 spin state consists of 8 CSFs that span the Hilbert space: {|*uud*⟩, |*udu*⟩, |*u*20⟩, |*u*02⟩, |2*u*0⟩, |0*u*2⟩, |20*u*⟩, |02*u*⟩}. Among them, the two CSFs^vvs^ which only contains open-shell couplings (*u* and *d*) form the van Vleck–Sherman subspace: {|*uud*⟩, |*udu*⟩}. The van Vleck–Sherman subspace can be conveniently represented graphically by the genealogical branching diagrams (see Figure 3 of ref 11 for examples and further details), which as opposed to the Shavitt diagrams, describe only the subspace of the unpaired electrons.

Accordingly, the GA fitness function may be restricted to the same subspace. The CSFs^vvs^ are the only CSFs that appear in Heisenberg models, whereas in ab initio systems dominated by exchange interactions, they form the subset of CSFs that contribute most significantly to the low-lying electronic states. Metal-to-metal and ligand-to-metal hopping are absent in the van Vleck–Sherman space, and while they are extremely important for the description of the low-energy states of exchange-dominated PNTM clusters, we have found them to be less important when designing an optimal GA fitness function. The number of CSFs^vvs^ is given by the formula^42^

3 $$N_{VVS}\left(N_{o},S\right)=\left(\frac{N_{o}}{N_{o}/2-S}\right)-\left(\frac{N_{o}}{N_{o}/2-S-1}\right)$$

where *N*_o_ and *S* refer to the number of singly occupied orbitals and the total spin, respectively.

For systems with local spin *S*_loc_ = 1/2, the CSFs^vvs^ describe the space of collinear spin states (magnetic sites with parallel or antiparallel spin arrangement), while for systems with local spin *S*_loc_ > 1/2, they count collinear and noncollinear spin states, and high-energy CSFs that violate the first Hund’s rule (maximum spin multiplicity on each magnetic site).

Since the Hamiltonian’s block-diagonal structure follows from eq 1 and is generally independent of the targeted spin state (a global property of the Hamiltonian), a site ordering that maximizes this structure for collinear states will also do so for noncollinear states. For systems with local spin *S*_loc_ > 1/2, it is sufficient to consider the smaller van Vleck–Sherman subspace of collinear states, whose dimensionality grows as for *S*_loc_ = 1/2 spin systems. Thus, it is independent of the number of unpaired electrons per magnetic site and it is possible to replace *N*_o_ with the number of magnetic sites, *N*_s_, in eq 3.

Despite the exponential scaling of the complete van Vleck–Sherman space, it is possible to conceive a computationally inexpensive genetic algorithm whose fitness function exclusively depends on the collinear CSFs^vvs^ (a smaller van Vleck–Sherman subspace). As an example, for the 8-site P-cluster with *S*_loc_ = 2 (4 unpaired electrons per site, 32 electrons in total), instead of a fitness function that searches over the entire van Vleck–Sherman space (containing a staggering number of 35 357 670 CSFs^vvs^), one limits the search only to the 14 collinear CSFs^vvs^. By this strategy, a 22-site system would require the evaluation of 58 786 CSFs^vvs^ elements to obtain the fitness score of a given site ordering.

The Hamiltonian matrix elements, including the CSF energies in the spin-adapted basis, can be evaluated using the GUGA. As we only consider CSFs^vvs^, only certain terms in the CSF energy expression (see eq 12 of Appendix A.1) are varying with the site ordering and the CSF of choice. Thus, as derived in Appendix A.1, the CSF energy evaluation for CSFs^vvs^ can be simplified as the following compact expression

4 $$\left\langle m\left|Ĥ\right|m\right\rangle =\sum_{i>j}g_{ij,ji}T_{ij}+const.$$

where *g*_*ij*,*ji*_ are (exchange) two-body integrals and const. absorbs the omitted constant terms, and

5 $$T_{ij}=\frac{{\left(-1\right)}^{d_{i}+d_{j}+1}}{2}\sqrt{\prod_{k=i}^{j-1}\frac{b_{k}-2d_{k}+4}{b_{k}+2d_{k}-2}\prod_{k=i+1}^{j}\frac{b_{k}+4d_{k}-5}{b_{k}+1}}$$

where *b*_*k*_ (twice the value of the cumulative spin, *s*_*k*_) and *d*_*k*_ (step-vector element) are GUGA specific parameters at the level *k*. More details on the GUGA framework and terminology can be found in various references (see, e.g., refs 18, 43–45 ).

Therefore, the computational cost for evaluating the CSF energies over only CSFs^vvs^ is further reduced by omitting terms that are identically zero or constant (const. in eq 4) across all CSFs^vvs^. By this formula, evaluating the CSF energies of the 58 786 CSFs^vvs^ takes approximately 27 s on a single core of an AMD EPYC 7763 processor (2.45 GHz), and as each CSF energy can be computed independently, this step is massively parallelizable.

We assessed the correlation between CSFs^vvs^ energies and the *L*_4_ norm of the wave function in six different cases with distinct Hamiltonians and physical characteristics, namely (a) an 8-site one-dimensional Heisenberg model with NN interactions (*J*_NN_ = 0.5 Ha), (b) an 8-site hydrogen chain (ab initio equivalent of (a)), with an interatomic distance of 3.6 Å, (c) an 8-site Heisenberg cluster model with site interactions inversely proportional to the interatomic distances, (d) an 8-site hydrogen cluster (ab initio equivalent of (c)), (e) an 8-site one-dimensional Heisenberg model with NN and next-NN interactions (*J*_2_/*J*_1_ = 0.5), and (f) an 8-site one-dimensional Heisenberg model with NN and next-NN interactions (*J*_2_/*J*_1_ = 1.0). For the one-dimensional chains, cases (a), (b), (e), and (f), open boundary conditions are applied. In cases (c) and (d), the cluster geometry reflects the positions of the metallic centers in the P-cluster of nitrogenase (see Figure 9).^46^ Cases (e) and (f) are designed to investigate the effect of frustration on the GA fitness function.

Table 1 shows the Pearson correlation coefficients between the *L*_4_ norm and the maximum and minimum CSF energies, and their differences

6 $$\Delta E_{diag}=\max_{|V\rangle }\left\langle V\left|Ĥ\right|V\right\rangle -\min_{|V\rangle }\langle V|Ĥ|V\rangle$$

evaluated over the 14 collinear CSFs^vvs^ (|*V*⟩) for the six different systems.

Max *E* (blue), Min *E* (orange), and Δ*E*_diag_ (green) are presented against 8!/8 = 5040 symmetrically nonequivalent site permutations^15^ (*x*-axis), for case (a) in Figure 2a and for case (b) in Figure 2b. Site permutations are sorted by increasing values of *L*_4_-norm.

**Table 1:** Pearson Correlation Coefficients between the *L*_4_ Norm and the Candidate Fitness Functions Based on the Diagonal Elements^a^

	correlation between *L*_4_ norm and:		
system	Max *E*	Min *E*	Δ*E*_diag_
(a) 8-site NN Heisenberg chain	0.3374	–0.8490	0.7404
(b) H_8_ chain (*r* = 3.6 Å)	0.8470	–0.3351	0.7379
(c) Heisenberg P-cluster (*J*_*ij*_ ∝ 1/*r*_*ij*_)	0.3926	–0.5079	0.4576
(d) H_8_ model of the P-cluster	0.6233	–0.4130	0.5102
(e) 8-site *J*_2_/*J*_1_ = 0.5 Heisenberg chain	0.4354	–0.8177	0.6537
(f ) 8-site *J*_2_/*J*_1_ = 1.0 Heisenberg chain	0.3145	–0.7742	0.5712

a When the correlation coefficient is 1 (−1), it indicates a perfect positive (negative) linear correlation, while a coefficient of 0 indicates no linear correlation.

**Figure 2:**
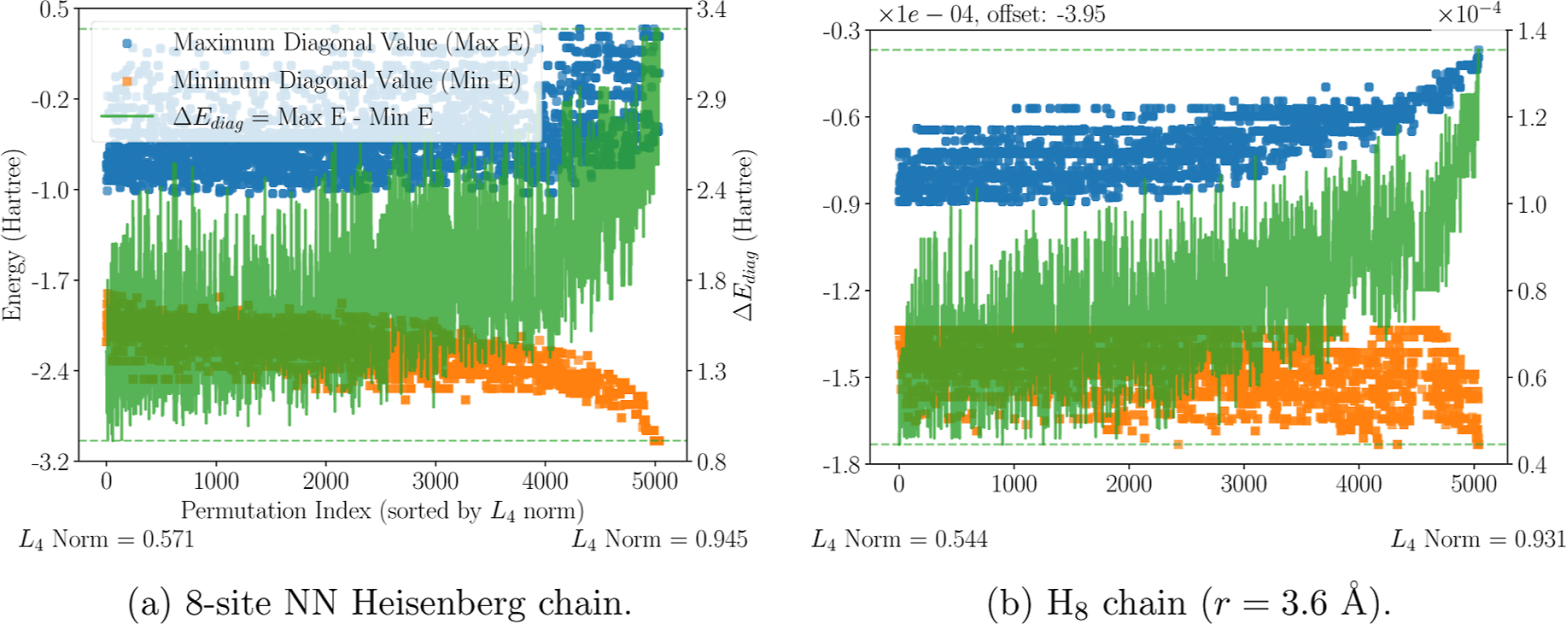
Lowest (yellow squares) and highest (blue circles) diagonal matrix elements, ⟨m|Ĥ|m⟩ , over the van Vleck–Sherman configuration space, and their difference (Δ*E*_diag_, green line) for two different 8-site chains. The left *y*-axis shows the value of the diagonal matrix elements, while the right *y*-axis shows the Δ*E*_diag_ value. The corresponding figures for cases (c–f) are reported in the Supporting Information (Figure S1).

Although Δ*E*_diag_ does not correlate strictly with the *L*_4_-norm, an overall moderate to strong correlation is observed in all cases. This trend is qualitatively consistent with the intuition that a larger spread in diagonal elements can lead to stronger localization of eigenfunctions in the standard basis, in line with the *majorization constraints* of the Schur–Horn theorem^47^ (see Section S3). This suggests that maximizing Δ*E*_diag_ over the collinear CSFs^vvs^ serves as an effective GA fitness function in the search of an optimal site ordering. Remarkably, the correlation between Δ*E*_diag_ and the *L*_4_ norm is also observed in the highly frustrated cases *J*_2_/*J*_1_ = 0.5 and *J*_2_/*J*_1_ = 1.0 (Figure S1e,f), suggesting that the Δ*E*_diag_-based fitness function remains effective for frustrated systems.

For the Heisenberg models (Figures 2a and S1c,e,f), a negative correlation is also observed between the lowest collinear CSF energy and the *L*_4_ norm. This supports the use of the lowest CSF energy as a fitness function in both the earlier SA approach^12^ and the present GA-driven strategy. In contrast, the highest diagonal matrix element shows a weaker correlation with the *L*_4_ norm, as evidenced by the smaller magnitudes of the correlation coefficients then those for the lowest CSF energy in Table 1 and many permutations with (near-)­degenerate highest diagonal values that span a wide range of *L*_4_ norms. For the ab initio systems (Figures 2b and S1d), we observe an almost opposite trend: the highest CSF energy correlates with the *L*_4_ norm, while the lowest exhibits a weaker correlation.

Thus, for Heisenberg models, the lowest CSF energy serves as a good proxy for wave function compactness, whereas for ab initio systems, the highest CSF energy is a better choice. However, since identifying one energy extreme requires scanning all diagonal terms for each permutation, using Δ*E*_diag_ as a fitness function does not increase the computational cost of the GA and remains a reasonable measure of wave function compactness for both Heisenberg and ab initio models.

The specular behavior of the lowest and highest diagonal matrix elements for Heisenberg models and ab initio systems in Figure 2 is a consequence of how exchange interactions are encoded into the matrix elements for the two types of Hamiltonians. On the basis of eq 4, Heisenberg models with antiferromagnetic NN interactions are characterized by negative exchange coupling parameters (*g*_*ij*,*ji*_ < 0); on the contrary, for ab initio systems, the exchange electron repulsion integrals, *g*_*ij*,*ji*_, are always positive, owing to their quadratic character. Thus, if for a given antiferromagnetic Heisenberg model, CSF |*A*⟩ is energetically more stable than CSF |*B*⟩, an inverted energy order is observed in the corresponding ab initio Hamiltonian with equivalent site ordering.

### 3.1 Strategy for Unfrustrated Systems

For unfrustrated systems, a further simplified fitness function can be used, which avoids searching for the extreme CSF energies and requires evaluating only a single *predetermined* diagonal matrix element for each site ordering. As reported in ref 12 and shown in Figure 2a, for unfrustrated Heisenberg models, the ordering with the lowest diagonal matrix element yields the most compact wave function. Hence, the simplified fitness function should incentivize orderings (and CSFs) giving large negative diagonal matrix elements.

Following eq 4, the nonconstant part of the diagonal matrix element for the NN Heisenberg Hamiltonian is

7 $$\sum_{i>j}J_{ij}\left(P\right)T_{ij}\left(|m\rangle \right)$$

where it is emphasized that *J*_*ij*_(*P*) and *T*_*ij*_(|*m*⟩) depend on the site ordering, *P*, and the CSF, |*m*⟩, respectively. For isotropic antiferromagnetic NN Heisenberg models, *J*_*ij*_(*P*) assumes the constant negative value *J* < 0, if the sites *P*(*i*) and *P*(*j*) are NNs, and 0 otherwise. In the case of a 6-site chain where the site labels are shown in Figure 3, when *P* = (1 – 3 – 5 – 2 – 4 – 6), *J*_*i*,*j*=1,2_ = 0 because site *P*(*i* = 1) = 1 and site *P*(*j* = 2) = 3 are not an NN pair. Whereas, *J*_*i*,*j*=2,5_ = *J* because sites *P*(*i* = 2) = 3 and site *P*(*j* = 5) = 4 are an NN pair.

**Figure 3:**
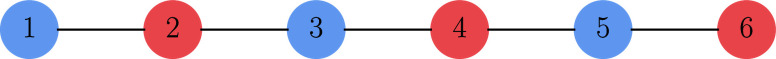
*S*–*M*_S_ mapping for a 6-site antiferromagnetic Heisenberg chain. The sites are labeled from 1 to 6. The *u* and *d* couplings are represented by blue and red circles, respectively.

Both *J*_*ij*_(*P*) and *T*_*ij*_(|*m*⟩) can be represented in matrix form. In this representation, **J**(*P*) acts as a weighting mask on **T**(|*m*⟩), so that only the unmasked elements of **T**(|*m*⟩) (corresponding to the nonzero entries of **J**(*P*)) contribute to the diagonal matrix element. This is visualized in Figure 4 of ref 12, where the unmasked elements of *T*_*ij*_ (denoted as *X*_*ij*_ up to a constant factor in that reference) are highlighted with black squares. Subfigure 4b of ref 12 corresponds to the example with *P* = (1 – 3 – 5 – 2 – 4 – 6) discussed in the previous paragraph.

Since the sign of *T*_*ij*_ is determined by its *sign factor*, ${\left(-1\right)}^{d_{i}+d_{j}+1}$ (eq 5), our goal is to choose a site ordering and a CSF such that the unmasked *T*_*ij*_ elements are positive. This condition is met when *d*_*i*_ ≠ *d*_*j*_, noting that *d*_*i*_ and *d*_*j*_ can only take values 1 (*u*, up-spin) or 2 (*d*, down-spin) in the Heisenberg model. Furthermore, the unmasked pairs (*i*, *j*) correspond to those for which *P*(*i*) and *P*(*j*) are nearest neighbors sites. Thus, unmasked positive elements are provided by assigning *u* and *d* couplings to sites in a pattern that ensures that no two neighboring sites carry the same coupling type. For example, in the 6-site chain this condition is satisfied by assigning *u* couplings (blue circles) and *d* couplings (red circles) to alternating sites, as shown in Figure 3.

Since the alternating pattern of *u* and *d* couplings resembles the Néel order where the spins of neighboring sites are antiparallel in a Slater determinant (SD) basis characterized by the total spin projection *M*_S_ quantum number, we refer to this as the *S*–*M*_S_ *mapping*. Under the *S*–*M*_S_ mapping, the CSF constructed from an ordering is referred to as the *S*–*M*_S_-*consistent CSF* of the ordering. For example, for the site ordering *P* = (1 – 3 – 2 – 5 – 4 – 6), the *S*–*M*_S_-consistent CSF is |*u*_1_ *u*_3_ *d*_2_ *u*_5_ *d*_4_ *d*_6_⟩. However, not all site orderings yield valid *S*–*M*_S_-consistent CSFs. For instance, the *S*–*M*_S_-consistent CSF of *P* = (1 – 2 – 4 – 3 – 5 – 6) is an invalid CSF, |*u*_1_ *d*_2_ *d*_4_ *u*_3_ *u*_5_ *d*_6_⟩, because it leads to a negative spin after cumulatively coupling the first three orbitals $\left(S_{k=3}=-\frac{1}{2}\right).$

Although in ref 12 we noted that Hamiltonian matrix elements can be evaluated in a matrix form as a sum over unmasked terms, the *S*–*M*_S_ mapping introduced here offers a systematic procedure for unfrustrated systems to select a unique *S*–*M*_S_-consistent CSF for any compatible site ordering, which ensures *d*_*i*_ ≠ *d*_*j*_ for adjacent sites and yields positive unmasked *T*_*ij*_ elements. This approach is crucial for the GA’s efficiency, as it simplifies the fitness function to a single CSF energy and further reduces the site permutation search space, in contrast to the earlier SA method, which explored the full van Vleck–Sherman space for each ordering.

Thus, for unfrustrated systems, we propose an inexpensive GA strategy in which: (a) the *S*–*M*_S_ mapping binds the orbital couplings (*u* and *d*) to the sites, and (b) only site orderings that yield valid *S*–*M*_S_-consistent CSFs (with positive cumulative spin values) are explored. Since this strategy employs a single *S*–*M*_S_-consistent CSF for each compatible ordering, the fitness function is simply given by the diagonal matrix element ⟨Ĥ⟩ of the *S*–*M*_S_-consistent CSF and is minimized during the GA optimization. This strategy enables the GA to find optimal orderings for large unfrustrated Heisenberg models (here up to a 44-site 2D-lattice), presented in Section 4.

This strategy is particularly effective, as it limits the GA search space only to those orderings that lead to valid *S*–*M*_S_-consistent CSFs. While the size of the full search space for a system with spin *S* and *N*_o_ spatial orbitals is *N*_VVS_(*N*_o_, *S*) × *N*_o_!, corresponding to the space explored in ref 12, here we restrict the search to site orderings that yield valid *S*–*M*_S_-consistent CSFs. Under this constraint, there are *N*_*u*_! × *N*_*d*_! orderings for each CSF, where *N*_*u*_ and *N*_*d*_ are the numbers of *u* and *d* entries, respectively, with *N*_*u*_ + *N*_*d*_ = *N*_o_ and *N*_*u*_ – *N*_*d*_ = 2*S*, and *N*_VVS_ valid CSFs, giving a total search space of size *N*_VVS_ × *N*_*u*_! × *N*_*d*_!. For example, in the 6-site singlet case, the original search space of 6! × 5 = 3600 (with 6! site orderings and 5 CSF^vvs^) is reduced to 5 × 3! × 3! = 180. In general, the reduction of the search space size amounts to a factor of

8 $$\frac{N_{VVS}\left(N_{o},S\right)\times N_{o}!}{N_{VVS}\left(N_{o},S\right)\times N_{u}!\times N_{d}!}=\left(\begin{array}{l} N_{o}\\ N_{d} \end{array}\right)$$

In ref 12, a *propagating doublet* along the chain was identified as the leading CSF in the site ordering that maximally compresses the wave function of a spin- $\frac{1}{2}$ antiferromagnetic nearest-neighbor Heisenberg chain, both with open and closed boundary conditions. In line with the above discussion, this CSF corresponds to the *S*–*M*_S_-consistent CSF of the site ordering *P* = (1 – 3 – 2 – 5 – 4 – 6) and is the lowest in energy among the CSFs^vvs^ (the rightmost orange marker in Figure 2a).

It is crucial to point out that the *S*–*M*_S_-consistent CSF does not always yield the lowest energy among all CSFs across all possible site orderings (see Figure S2). This is because, although the *S*–*M*_S_ mapping ensures that the nonzero terms in eq 7 are negative, it does not guarantee that the unmasked *T*_*ij*_ elements have large magnitudes. By also considering the magnitudes of the *T*_*ij*_ elements, one can gain further insight into the CSF structures that minimize the diagonal matrix elements for any given site ordering. In the present work, we have not exploited the magnitudes of the *T*_*ij*_ elements, as the *S*–*M*_S_-consistent CSF alone suffices to obtain a compact wave function in unfrustrated systems. Further discussion of the factors that maximize the magnitude of the unmasked *T*_*ij*_ elements is provided in Appendix A.2.

Beyond the NN scenario, **J**(*P*) is no longer a simple mask with only 0 and constant *J* entries, but a matrix featuring entries of varying magnitude and sign. In such cases, assigning *u* and *d* couplings to ensure positive unmasked *T*_*ij*_ elements is not always possible, rendering the *S*–*M*_S_ mapping inapplicable.

## 4 GA Application to NN Heisenberg Lattice Models

In this section, we apply the GA to various unfrustrated antiferromagnetic NN Heisenberg lattice models. The fitness function based on the *S*–*M*_S_-consistent CSF, introduced in Subsection [Sec sec3.1], is adopted. We report the optimal site orderings obtained for lattice models with up to 44 sites. Results for 4 × 4, 5 × 5, and 6 × 6 square lattices are presented in Section S5 in the Supporting Information.

### 4.1 20-Site Chain

As a validation, we apply the GA strategy with the simplified fitness function to a 20-site NN Heisenberg chain, which ref 12 identifies as having the optimal site ordering (1 – 3 – 2 – 5 – 4 – 7 – 6 – 9 – 8 – 11 – 10 – 13 – 12 – 15 – 14 – 17 – 16 – 19 – 18 – 20). Using a GA with 200 chromosomes per generation, and a mutation rate of 0.05, the best ordering is found after 488 generations, exploring fewer than 97 600 permutations, which is vastly smaller than the full search space of 16 796 × 10! × 10! ≈ 2.2 × 10^17^ permutations allowed by the *S*–*M*_S_ mapping.

Figure 4 summarizes results on the sensitivity of the GA on mutation rate (*m*) and CS period. Population size (200 chromosomes) was kept fixed, while varying *m* over 0.00 (orange), 0.05 (green), and 0.15 (red), and using CS periods of 2 (dashed lines) and no CS (solid lines). Additionally, simulations with *m* = 1.00 (blue) were performed, where non-elite chromosomes are randomly generated at each generation. For each parameter set, the GA was executed 100 times with different seeds (random initial populations) for statistical analysis.

**Figure 4:**
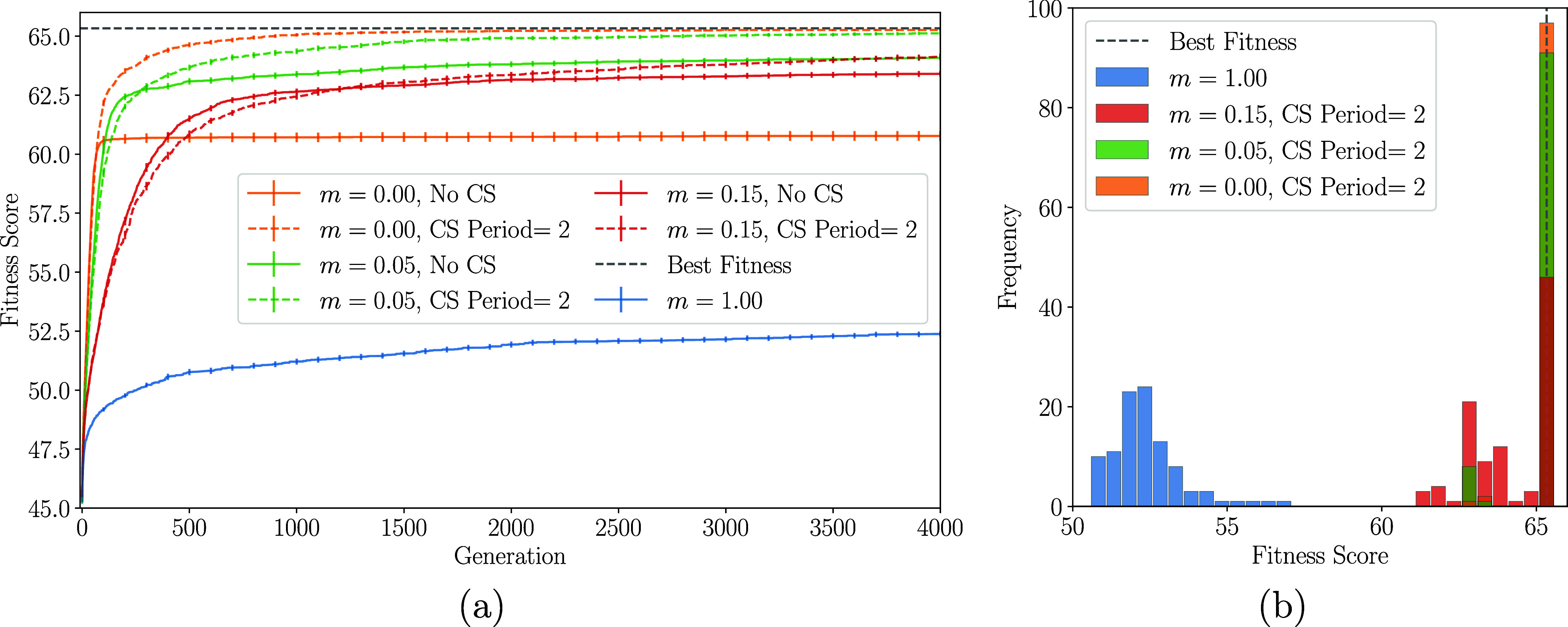
(a) Average best fitness score over the 100 GA simulations using different mutation rates (*m*) and CS periods. (b) Histogram of the best fitness scores of the 100 independent simulations at generation 4000 for each set of parameters. Only CS periods of 2 are shown for clarity. The dashed gray lines indicate the highest fitness score.

Figure 4a shows the evolution of the mean best fitness across 100 independent GA runs over 4000 generations. Highly randomized simulations (*m* = 1.00) evolve noticeably slower than others, indicating that inheritance from parent chromosomes is essential for efficient search-space exploration. Conversely, fully deterministic runs without mutation (*m* = 0.00) and without CS converge quickly initially but stagnate later, lacking the random variation needed to escape local minima. GA simulations with moderate randomness evolve faster, suggesting that mutation rate and CS period balance Monte Carlo-style exploration and GA-driven inheritance. Optimal performance lies between extremes: excessive randomness slows convergence, while too little randomness restricts exploration and causes the algorithm to get trapped in local minima. For each mutation rate *m*, the CS period 2 (dashed line) initially shows slower improvement compared to no CS, but eventually surpasses it. The shuffling period 2 without mutation (*m* = 0.00) delivers the best performance among all tested parameters, with average fitness scores approaching the maximum possible value (dashed gray horizontal line), which correspond to the propagating doublet site ordering. Figure 4b shows the histogram of the best fitness scores from 100 independent GA runs at generation 4000. The data confirm that MC-type simulations (blue) produce significantly lower best fitness scores than other GA variants.

### 4.2 2 × 11, 3 × 11, and 4 × 11 Ladders

Next, we applied the GA to *N*-leg ladders, for which optimal orderings have not yet been identified. A GA simulation on a 2 × 11 lattice (100 chromosomes per generation) converged after 620 generations to the ordering (1 – 13 – 12 – 3 – 2 – 15 – 14 – 5 – 4 – 17 – 16 – 7 – 6 – 19 – 18 – 9 – 8 – 21 – 20 – 11 – 10 – 22) and the reference CSF |*uududududududududududd*⟩ (Figure 5). The GA explored fewer than 62 000 permutations, vastly smaller than the restricted search space size of 9.3 × 10^19^. Consistent with our analysis (eq 25, Appendix A.2), many alternating *ud* couplings follow the initial increase in *b*_*k*_ set by the first *u* coupling. The ordering prioritizes traversing nearby sites to minimize the total *orbital-index difference* |*j* – *i*|, as discussed in Appendix A.2, of unmasked terms in eq 7.

**Figure 5:**
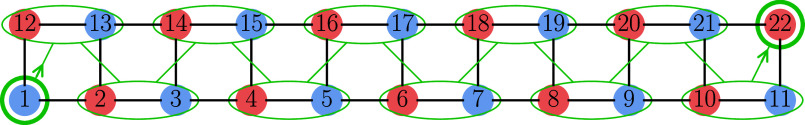
2 × 11 ladder with the *S*–*M*_S_ mapping. Blue and red circles denote *u* and *d* spin couplings, respectively, with site labels indicated. Green lines trace the GA-optimized ordering, starting at the first *u* coupling (site 1) and ending at the last *d* coupling (site 22). Thick green circles mark the start and end of the sequence. A green ellipse represents a *ud* coupling, thus the corresponding ordering sequence sweeps across the blue circle and then the red circle.

We performed FCIQMC calculations with 50 000 walkers using four different site orderings: (a) the *natural* ordering: (1 – 2 – 3 – 4 – 5 – 6 – 7 – 8 – 9 – 10 – 11 – 12 – 13 – 14 – 15 – 16 – 17 – 18 – 19 – 20 – 21 – 22), (b) the snake-like ordering (*snake 1*): (1 – 2 – 3 – 4 – 5 – 6 – 7 – 8 – 9 – 10 – 11 – 22 – 21 – 20 – 19 – 18 – 17 – 16 – 15 – 14 – 13 – 12), (c) the snake-like ordering (*snake 2*): (1 – 12 – 13 – 2 – 3 – 14 – 15 – 4 – 5 – 16 – 17 – 6 – 7 – 18 – 19 – 8 – 9 – 20 – 21 – 10 – 11 – 22), and (d) the GA-optimized ordering.

Table 2 summarizes the leading CSF energies and weights, the projected energies for each ordering, and the DMRG benchmark energy with bond dimension *M* = 1000. The leading CSFs of the *snake 2* and *GA* orderings are |*ududududududududududud*⟩ and |*uududududududududududd*⟩, respectively; these represent the *S*–*M*_S_-consistent CSFs in each case.

**Table 2:** Leading CSF Energies, Their Weights, and Projected Energies for the 2 × 11 Ladder with Four Different Site Orderings

ordering	leading CSF energy (Ha)	leading weight (%)^b^	projected energy (Ha)^c^
natural	–1.759	0.5	unstable
snake 1	–1.417	2.3	unstable
snake 2	–16.500	14.4	–24.779 ± 0.012
GA	–22.889	71.3	–24.766 ± 0.002
DMRG[Table-fn t2fn4]			–24.767

a Instantaneous FCIQMC wave function weights.

b Blocking analysis^48^ over 4000 iterations.

c The DMRG benchmark energy is included for comparison. *M* = 1000.

The *GA* ordering results in the lowest reference CSF energy and the largest leading CSF weight, indicating that the corresponding many-body wave function is highly compact. The *natural* and *snake 1* orderings lead to highly unstable FCIQMC dynamics, as the walker number is insufficient to overcome the sign problem.^49^ In contrast, the *snake 2* and *GA* orderings yield stable FCIQMC dynamics (Figure 6).

**Figure 6:**
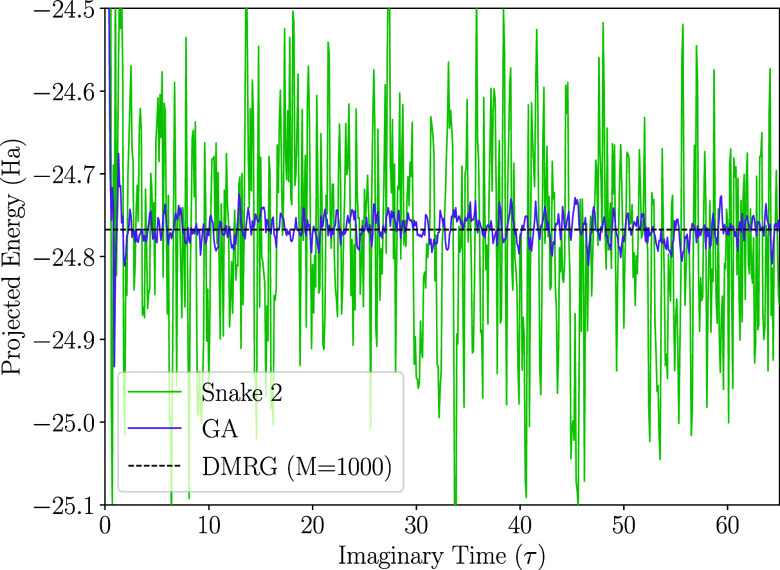
Projected energies from FCIQMC simulations on the 2 × 11 ladder using the *GA* and *snake 2* orderings. The dashed horizontal line marks the DMRG benchmark energy.

The *snake 2* ordering performs better than the *natural* and *snake 1* cases but remains inferior to the *GA* ordering. Its large error-bar renders the projected energy of *snake 2* unsuitable for practical applications and requires longer dynamics to reduce statistical uncertainty. In contrast, the *GA* ordering yields more accurate and precise results, reflecting the more compact nature of the wave function. The standard error of the *GA* ordering is 2 mHa, which is 6 times smaller than that of the *snake 2* ordering, 12 mHa, and the projected energy agrees with the DMRG benchmark within the estimated error.

Notably, the FCIQMC simulations on lattice models presented here were performed without the initiator approximation.^6–8^ In the absence of the initiator approximation, all determinants can spawn walkers freely, often leading to a rapidly growing noise-to-signal ratio. This is a manifestation of the sign problem in FCIQMC, which can only be suppressed by increasing the total number of walkers (typically by orders of magnitude) rendering noninitiator FCIQMC generally impractical. When the walker population exceeds a system-dependent threshold, known as the *annihilation plateau*,^49^ the desired wave function emerges over the course of the simulation. Owing to the quasi block-diagonal structure of the Hamiltonian matrix, which reduces the effective Hilbert space size, the GA ordering lowers the annihilation plateau and enables stable FCIQMC dynamics even at walker populations insufficient for nonoptimal orderings.

The application of the GA simulation to the 3 × 11 (500 chromosomes per generation) and 4 × 11 (2000 chromosomes per generation) ladders identified the ordering and leading CSFs as shown in Figures 7 and 8, respectively.

**Figure 7:**
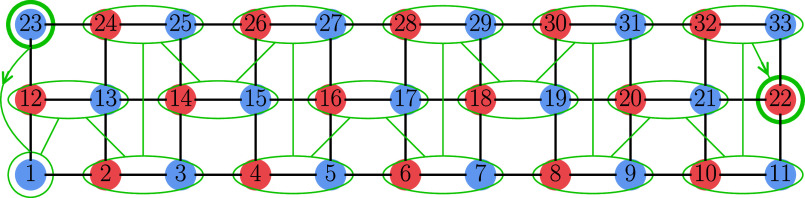
3 × 11 ladder with the GA-found optimal ordering and the *S*–*M*_S_-consistent CSF. Color coding and site labels are as in Figure 5.

**Figure 8:**
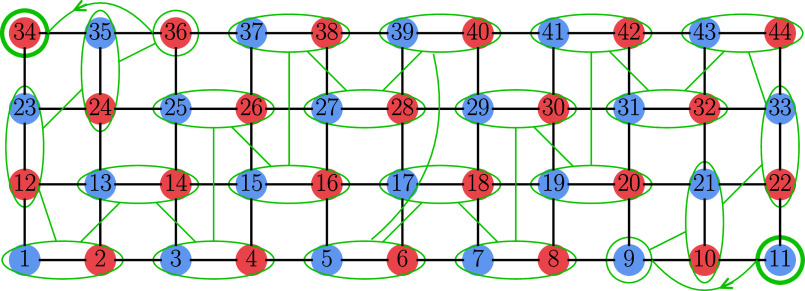
4 × 11 ladder with the GA-found optimal ordering and the *S*–*M*_S_-consistent CSF. Color coding and site labels are as in Figure 5.

In contrast to the 2 × 11 case, which starts with a single *u* coupling, for the 3 × 11 and 4 × 11 lattices, the GA suggests orderings starting with two *u* couplings followed by *ud* pairs. This reflects the larger overall coordination numbers of the 3 × 11 and 4 × 11 lattices, which result in larger orbital-index differences. This increases the number of internal factors in eq 5, amplifying the reduction of *T*_*ij*_ magnitudes when *b*_*k*_ is small. Thus, while the one-dimensional Heisenberg model favors a *propagating doublet*, in two-dimensional systems (e.g., ladder-like 4 × 11 or square 6 × 6 lattices), the optimal structures resemble a *propagating triplet*, with the length of the initial consecutive *u* couplings scaling with the lattice coordination number.

## 5 GA Application to Ab Initio Models

For ab initio systems, the *S*–*M*_S_ mapping strategy is generally not applicable, as in general a Néel-like reference state cannot be reliably predicted. In these cases, we employ the more general GA strategy described in Section 3, which maximizes the Δ*E*_diag_ between the largest and smallest diagonal elements among collinear CSFs^vvs^. This multi-CSF fitness function is computationally more demanding, but the restriction to collinear CSFs^vvs^ limits the search space sufficiently for the GA to converge within a manageable number of generations while keeping the overall cost low.

We demonstrate the applicability of the general GA strategy on three ab initio systems: (a) the hydrogen cluster H_8_ in which the hydrogen atoms are placed at the Fe sites of the P-cluster, with an active space comprising the eight 1s orbitals and their electrons, CAS­(8,8); (b) the neutral P-cluster model (P^*N*^, Figure 9), with an active space consisting of the 8 × 5 3d valence orbitals of the eight Fe­(II) centers and their 8 × 6 electrons, CAS­(48,40); and (c) the same P^*N*^ model with a larger active space, CAS­(114,73), which includes bridging and peripheral sulfur 3p orbitals and electrons beyond CAS­(48,40), thereby enabling us to reuse the ordering obtained for (b), owing to the unchanged diagonal elements. Details on these models are provided in Section S1.

**Figure 9:**
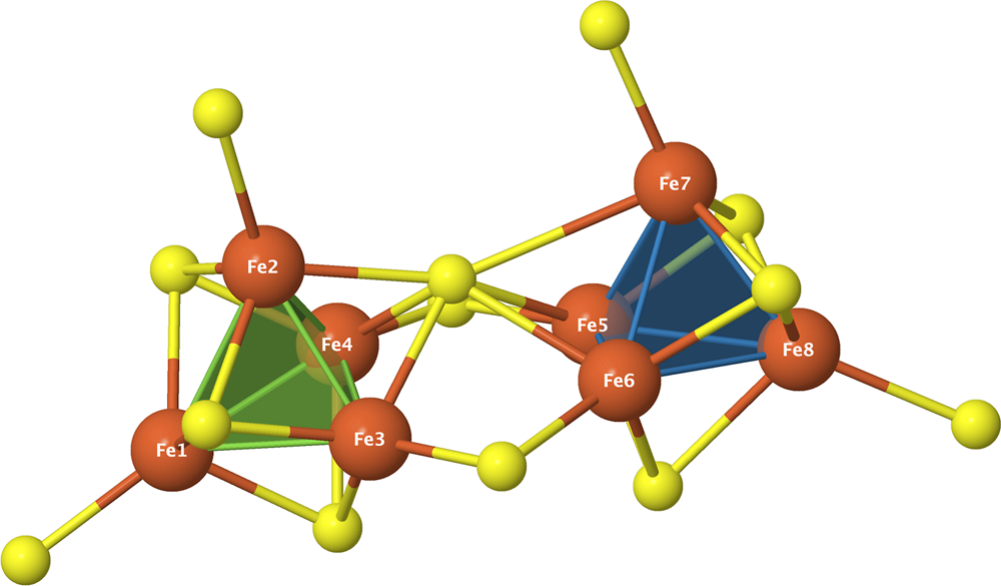
P-cluster in its neutral state, P^*N*^. Fe atoms are shown in orange, S atoms in yellow. Other atoms omitted from the figure for clarity. Labels are indicated for the magnetic sites carrying the unpaired electrons. The P^*N*^-cluster can be described as two cubane-like structures sharing a central S atom (corner-shared), with two additional peripheral S atoms bridging the two cubanes. The Cartesian coordinates of the P-cluster are taken from ref 46.

The first system serves as a validation case, allowing exact diagonalization over all 8! = 40 320 site orderings. The other examples are too large for exact diagonalization for all site orderings; here, the GA performance is assessed a posteriori by showing that its suggested ordering yields stable FCIQMC dynamics, unlike lower-fitness alternatives.

In earlier studies, we observed that site orderings maximizing wave function compression of minimal active spaces—comprising only magnetic orbitals and unpaired electrons—also compress wave functions in enlarged active spaces that include ligand orbitals and their electrons. For instance, site orderings compressing the CAS­(10,10) of the all-ferric Fe­(III)_2_S_2_ and the CAS­(20,20) of the Fe­(III)_4_S_4_ clusters also compress the larger CAS­(22,26)^9^ and CAS­(44,32)^10^ wave functions, respectively, for ground and excited states. Similarly, the ordering compressing the CAS­(9,9) of a Mn­(IV)_3_O_4_ system also compresses the larger CAS­(55,37) wave function.^11^ Finally, in ref 14, the ordering compressing the CAS­(12,12) of a Co­(II)_3_Er­(III)­(OR)_4_ cubane also compresses its CAS­(56,56) wave functions across all low-energy spin states. Consistent with these findings, we apply the ordering compressing the CAS­(48,40) wave function of the P-cluster to the larger CAS­(114,73) over the lowest states of the 14 *selected* collinear singlet states.

### 5.1 H_8_ Cluster Model

The exact diagonalization of the CAS­(8,8) for all 8! orderings of H_8_ reveals the best orderings, characterized by a ground state *L*_4_ norm of 0.955571, and the worst orderings, characterized by an *L*_4_ norm of 0.572039. The fitness scores (Δ*E*_diag_) of the best and the worst orderings are 0.001169 and 0.000463, respectively (Figure S1d). A GA simulation using 20 chromosomes per generation and a mutation rate of 0.05, yields the best ordering, (3 – 2 – 1 – 4 – 7 – 8 – 6 – 5), after only 2 generations. This ordering first sweeps the magnetic sites in one tetrahedron and then the other (the H atoms follow the same magnetic site labels as the Fe atoms in the P-cluster shown in Figure 9). The Δ*E*_diag_ fitness score and the *L*_4_ of this ordering correspond to the best possible values. The corresponding ground state wave function is highly compact, being dominated by the single CSF |*udududud*⟩ that has a weight of 95.5%.

### 5.2 P-Cluster Model

All systems investigated by the GA so far feature local spin- $\frac{1}{2}$ . In contrast, the neutral P-cluster is best described as a local spin-2 system, with four unpaired electrons per site. Previous studies have shown that permutations of singly occupied orbitals *within* individual magnetic centers do not affect wave function compression and can therefore be safely ignored during the GA search. Furthermore, the orbitals used for the GA search and FCIQMC dynamics were obtained from ROHF optimization targeting total spin *S* = 16, ensuring a clear separation between doubly and singly occupied orbitals. Orbital localization was then performed separately on these two sets, preserving their occupation character (an energy invariant transformation). This protocol introduces a controlled bias that favors the localization of electron pairs on the same orbital within each metal center (the doubly occupied ones in ROHF). As a result, in the GA search the electronic configuration at each Fe site was restricted to *U* = 2*uuuu* and *D* = 2*dddd*, reducing the search space to 8! site permutations instead of 40!

Given the manageable size of the search space (8!), we performed an exhaustive evaluation of Δ*E*_diag_ for all possible orderings. This allowed us to (i) assess the GA’s ability to identify (near-)­optimal orderings, and (ii) systematically analyze how orderings with varying fitness scores affect subsequent FCIQMC dynamics. With a Δ*E*_diag_ = 0.01847 fitness score, the GA converged to the (3 – 4 – 2 – 1 – 8 – 7 – 5 – 6) ordering, the globally optimal ordering as confirmed by the exhaustive search.

The impact of site ordering on spin-adapted wave function compactness is illustrated by comparing four orderings with distinct fitness scores, evaluated through their effect on FCIQMC stability: (a) the GA-optimized ordering for the actual P-cluster (*Fe*_*8*_ *-GA ordering*); (b) the ordering obtained by applying the GA to the H_8_ cluster model (*H*_*8*_ *-GA*); (c) an ordering with an intermediate fitness score (*intermediate ordering*); and (d) the ordering with the lowest fitness score (*bad ordering*). The corresponding fitness scores are listed in Table 3 along with the used site orderings.

**Table 3:** Fitness Scores (Δ*E*_diag_) for the Four Tested Orderings

ordering	magnetic site ordering	fitness score
Fe_8_-GA	(3 – 4 – 2 – 1 – 8 – 7 – 5 – 6)	0.01847
H_8_-GA	(3 – 2 – 1 – 4 – 7 – 8 – 6 – 5)	0.01835
intermediate	(2 – 4 – 5 – 1 – 8 – 7 – 3 – 6)	0.00961
bad	(3 – 6 – 7 – 2 – 1 – 8 – 4 – 5)	0.00429

#### 5.2.1 The Medium Active Space, CAS­(48,40)

For each of the four orderings, we performed i-FCIQMC calculations on the CAS­(48,40) active space, targeting the lowest-energy state for each of the 14 collinear singlet states. A walker population of 1 million sufficed to distinguish effective from ineffective orderings and to energetically resolve the 14 states (Figure 10).

**Figure 10:**
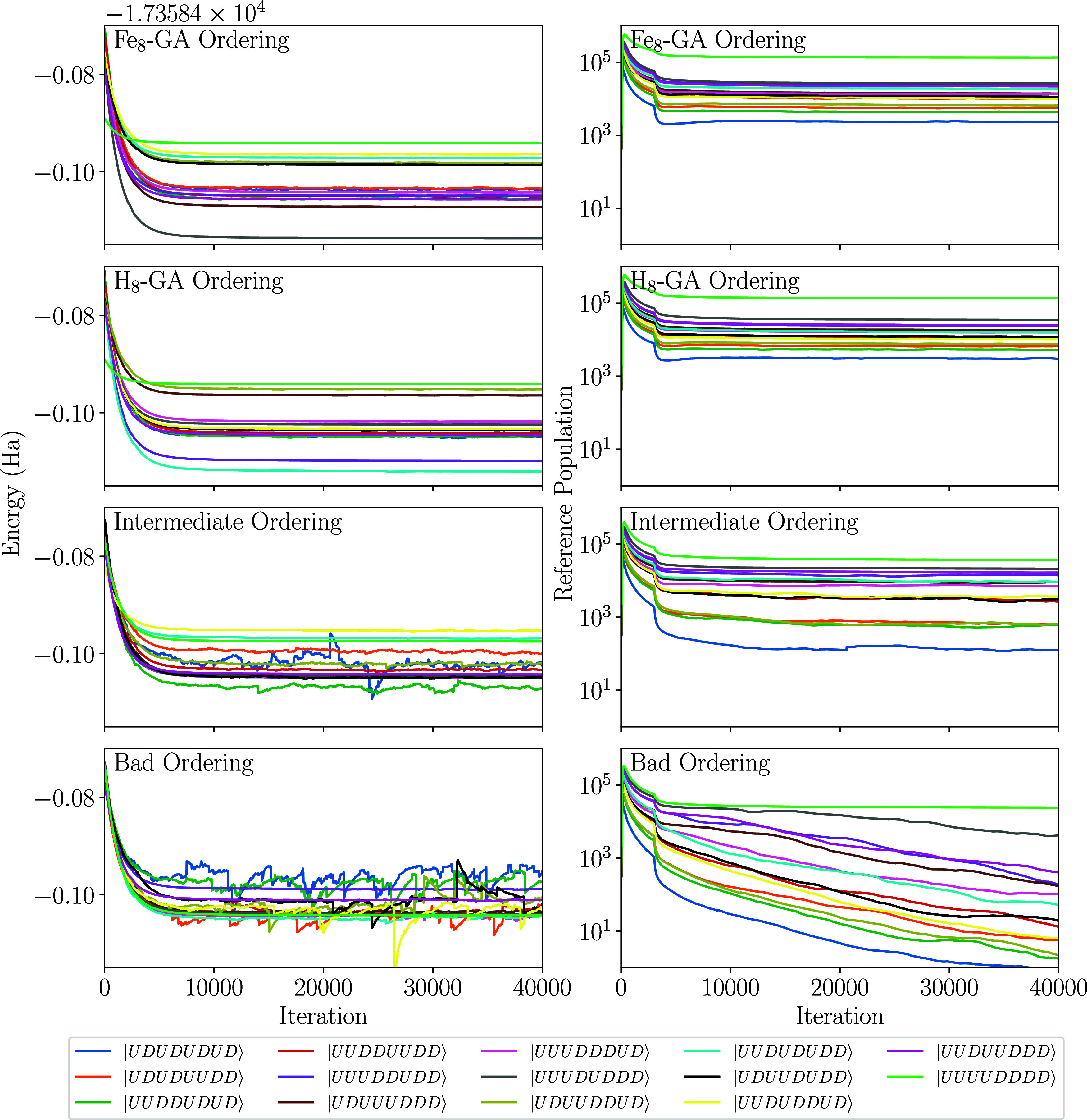
Projected energies (left) and reference CSF populations (right) as a function of FCIQMC iterations for the 14 lowest collinear singlet states of the CAS­(48,40) active space for the P^*N*^ cluster, using four different site orderings.

As expected, the overall noise in the projected energy is lowest for the *Fe*_8_ *-GA* ordering and increases as the ordering’s fitness score decreases. The noise for the *intermediate* ordering is already too high preventing the separation of some collinear singlet spin states. The noise correlates directly with the walker population on the reference CSF: while the *Fe*_8_ *-GA* ordering maintains a high and stable reference population, lower-fitness orderings yield progressively reduced populations. The *bad* ordering is particularly critical, showing a steady decline in reference population—a clear sign that the walker number is insufficient to sustain stable dynamics. At such low reference populations, the FCIQMC dynamics becomes noise-dominated, preventing resolution of the 14 collinear states and even obscuring the qualitative characterization of the electronic ground state. These results underscore the critical role of optimal site ordering in FCIQMC, enabling stable dynamics with fewer walkers than nonoptimal choices.

The 14 states examined here are not necessarily the 14 lowest-energy singlet states of the system; rather, they are the *selectively targeted* lowest-energy *collinear* singlet states. Noncollinear states may be energetically close to, or even lower than, the targeted collinear states; however, they are excluded by the choice of trial configurations used to initiate FCIQMC, due to the block-diagonal structure imposed by Quantum Anamorphosis when an optimal ordering is used. This selectivity facilitates the sampling of specific states of interest and distinguishes our approach from other methods. Less optimal orderings lead to denser Hamiltonian matrices, which hinder such selectivity, the dynamics become unstable, and the character of the 14 targeted states can no longer be reliably assessed.

We also observe that within the i-FCIQMC simulations, the optimal ordering significantly reduces the initiator error arising from the truncation of the Hilbert space. The (quasi-)­block-diagonal structure of the Hamiltonian induced by the optimal ordering mitigates the truncation, enabling accurate energy estimates even at lower walker populations. In the *Fe*_8_ *-GA* ordering, each of the 14 collinear states is highly dominated by a single CSF (see Table S1), leaving little opportunity for the initiator truncation to introduce significant errors. Exemplarily, the *Fe*_8_ *-GA* ordering yields a lower ground-state energy (gray |*U*_3_ *U*_4_ *U*_2_ *D*_1_ *U*_8_ *D*_7_ *D*_6_ *D*_5_⟩ signal in Figure 10) compared to other orderings, while maintaining a much higher reference population.

Table 3 shows that the *H*_8_ *-GA* ordering has a fitness score very close to that of the *Fe*_8_ *-GA* ordering, suggesting that, although not best, it may still serve sufficiently well in compressing collinear ground- and excited-state wave functions and in supporting stable FCIQMC dynamics. The comparison of the two orderings suggests that it might be generally possible to use simpler, H-like clusters, to identify good orderings for larger, more realistic PNTM systems. Nonetheless, this strategy does not guarantee a globally optimal solution, and transferring site orderings from one chemical model to another may compromise the final compression effect. A direct GA search on the system of interest is therefore recommended to ensure the best possible wave function compression.

#### 5.2.2 The Large Active Space, CAS­(114,73)

The inclusion of ligand orbitals in the larger CAS­(114,73) active space does not affect the fitness scores we have used, as the diagonal elements remain unchanged. Consequently, the optimal *Fe*_8_ *-GA* ordering identified for the CAS­(48,40) space is directly transferable to CAS­(114,73). Using this ordering, we performed FCIQMC calculations on CAS­(114,73), targeting the same 14 collinear states. A walker population of 10 million was employed for each state.

The resulting projected energies are summarized and compared to the CAS­(48,40) results in Table S1. The comparison shows slight decreases in the leading CSF weights of the CAS­(114,73) due to the increased Hilbert space size for the presence of metal-to-ligand hopping configurations in the wave functions. However, the leading CSFs remain dominant, suggesting that the wave functions of the collinear states are still highly compact.

Figure 11 shows the energy level diagram for CAS­(48,40) and CAS­(114,73) active spaces of the P-cluster. Inclusion of the ligand orbitals leads to increased energy gaps between the lowest collinear state (gray) and the remaining 13 collinear states. While some states exchange their relative stability compared to the CAS­(48,40) case, the lowest and highest energy states remain unchanged.

**Figure 11:**
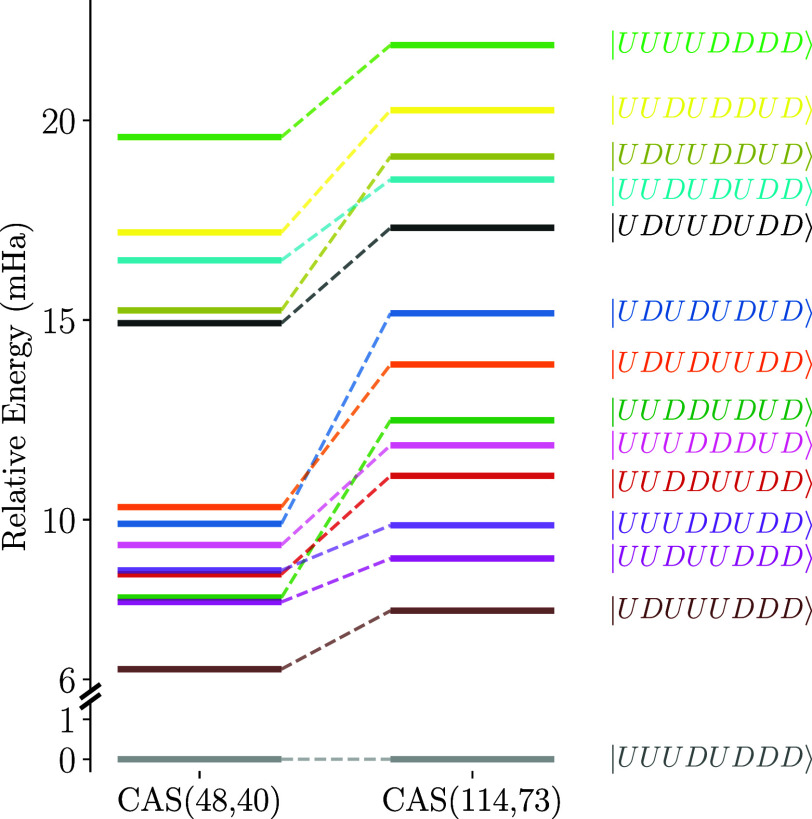
Energy level diagram of the 14 collinear singlet states for CAS­(48,40) and CAS­(114,73) of the P-cluster. The energies are shifted such that the lowest collinear state energy is set to zero.

Our results on the P-cluster model clearly show that the GA-derived ordering effectively compresses the wave functions of all 14 collinear states of interest simultaneously. In a previous study^10^ on the Fe­(III)_4_S_4_ cluster, we similarly observed that a single ordering could compress the wave functions of all low-energy spin states, regardless of whether they were collinear or noncollinear. However, in general a single orbital ordering is not optimal for qualitatively different electronic states, for example differing in the number of magnetic orbitals. For instance, charge-transfer states introduce new singly occupied orbital on the sulfur atoms, creating additional magnetic centers. While for the neutral exchange-coupled states the S 3p orbitals can be safely excluded from the GA search and listed after the magnetic orbitals, as we did for the CAS­(114,73) of the P-cluster model, this approach is no longer valid for charge-transfer states. Instead, the S-centered magnetic orbitals should be included in the GA search, which may lead to an orbital ordering that is optimal for the charge-transfer state but suboptimal for the neutral states.

## 6 Conclusions

We have developed a genetic algorithm (GA) to identify optimal orbital orderings that yield highly compact representations of both ground- and excited-state wave functions in spin-adapted bases.

Critically, we introduced computationally inexpensive fitness functions that serve as effective proxies for wave function compactness, and provided a GA framework to efficiently explore the site permutation space. In general, maximizing the gap between the largest and smallest collinear CSFs^vvs^ energies proves to be a practical fitness criterion across a broad range of systems, including both unfrustrated and frustrated Heisenberg lattice models, as well as challenging ab initio systems with many unpaired electrons. Moreover, for unfrustrated systems, we devised a strategy that reduces the CSF space to a single *S*–*M*_S_-consistent CSF energy, significantly lowering the computational cost. The *S*–*M*_S_ mapping further restricts the permutation space by binding each ordering to a valid CSF, thereby eliminating orderings that yield invalid CSFs from the search.

The performance of the GA—assessed in terms of convergence speed—is influenced by the degree of randomness in the simulation, governed by the mutation rate and the CS period. Insufficient randomness leads to stagnation, whereas excessive randomness hampers convergence. Optimal performance is therefore achieved at an intermediate level of randomness, balancing exploration and exploitation.

For small systems, where exhaustive search is feasible, we have shown that the GA converges to the known optimal ordering after sampling only a small fraction of the total search space. For larger lattice systems, including *N*-leg ladders and square lattices with up to 44 sites—for which no optimal orderings have previously been reported—the GA identifies orderings that are likely to be optimal, as evidenced by the high stability of spin-adapted FCIQMC dynamics and significantly reduced variance.

Suboptimal orderings lead to unstable FCIQMC dynamics, owing to the FCIQMC sign problem, thereby preventing the extraction of meaningful results. In contrast, optimal orderings yield stable dynamics at the same walker population, resulting in clear qualitative improvements and enhanced computational efficiency. For example, the GA-derived ordering resolves the 14 energetically lowest collinear singlet states within the large CAS­(114,73) for the P^*N*^ state of the P-cluster using only 10^7^ walkers—a population traditionally deemed insufficient for stable FCIQMC dynamics in such large active spaces and strongly correlated systems. Compared to the smaller CAS­(48,40), the extended CAS­(114,73) reveals differential stabilization of the lowest collinear states via a ligand-mediated superexchange mechanism involving the bridging atoms. Our GA-enhanced FCIQMC dynamics indicate a singlet ground state characterized by *S*_A_ = *S*_B_ = 4 local spin states on each of the two cubanes, with antiferromagnetic alignment across the two units, in agreement with the available literature.^46^

The GA-driven Quantum Anamorphosis represents a powerful and versatile tool for addressing strongly correlated, many-unpaired-electron systems. The quasi block-diagonal Hamiltonian structure achieved by our approach enables the selective targeting of the energetically lowest collinear states, rather than the global lowest-energy states. This feature offers clear advantages for sampling specific collinear states. This capability sets our approach apart from other excited-state methods. It could be advantageous in the design of novel materials, including polynuclear transition metal clusters relevant to biomimetic catalysis, advanced magnetic systems, and photoactive spin-switchable materials, where specific electronic states are prime target.

Although demonstrated here in the context of FCIQMC, the GA-driven Quantum Anamorphosis naturally generalizes to other electronic structure methods that approximate full-CI solutions via Hilbert space truncation, such as the selected-CI family of methods^50–57^ when formulated in spin-adapted bases.

Furthermore, given the rapid convergence of FCIQMC within the GA-driven Quantum Anamorphosis framework at remarkably low walker populations, and reduced wall-clock time, we envision the development of a protocol to generate high-quality training data for machine learning models aimed at constructing ab initio-quality force fields for molecular dynamics simulations under realistic conditions, even for systems with a large number of unpaired electrons.

Finally, we regard the GA-driven Quantum Anamorphosis as a promising framework for developing (1) machine learning models with reduced training complexity, and (2) quantum algorithms with reduced circuit depth, enabling more efficient electronic state representations. To date, quantum algorithms have primarily been formulated in spin-nonadapted bases, where spin symmetry is approximated using the angular momentum addition theorem.^58–61^ More recently, quantum algorithms have been designed to operate directly in the eigenbasis of the total spin operator, leveraging either the unitary group approach (UGA)^62^ or the symmetric group approach (SGA).^63^ The GA-driven Quantum Anamorphosis framework is particularly valuable here, since its compact wave function representations reduce sensitivity to algorithmic approximations and quantum decoherence. For example, in ref 63, a truncation scheme was introduced for intermediate spin couplings. Under the Quantum Anamorphosis framework, such truncation errors may become negligible, as the omitted intermediate couplings carry minimal weight in the compact wave function.

## Supplementary Material





## Figures and Tables

**Figure 12:**
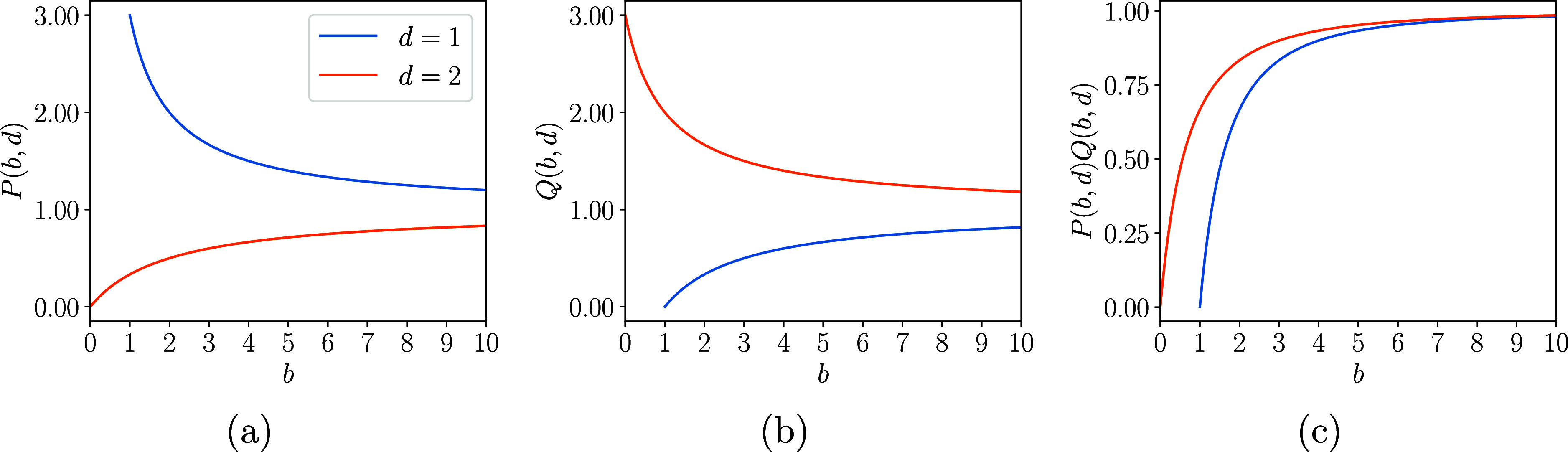
(a) *P*(*b*, *d*), (b) *Q*(*b*, *d*), and (c) *P*(*b*, *d*)*Q*(*b*, *d*) over various *b* values for *d* = 1 (blue) and *d* = 2 (orange).

## A Appendix

### A.1 Simplified Evaluation of ⟨m|Ĥ|m⟩

Given a CSF^vvs^, |*m*⟩, its diagonal element over the general spin-free Hamiltonian Ĥ can be expressed as

9
$$\left\langle m\left|Ĥ\right|m\right\rangle =\sum_{i,j}h_{ij}\left\langle m\left|{Ê}_{i,j}\right|m\right\rangle +\frac{1}{2}\sum_{i,j,k,l}g_{ij,kl}\left\langle m\left|{ê}_{ij,kl}\right|m\right\rangle$$

The first *coupling term*, $\left\langle m\left|{Ê}_{i,j}\right|m\right\rangle$ , vanishes for any hopping between two orbitals/sites, *i* ≠ *j*, while for *i* = *j*, the coupling term is equal to the occupation number of the orbital *i* in the CSF |*m*⟩

10
$$\left\langle m\left|{Ê}_{i,j}\right|m\right\rangle =\{\begin{array}{ll} N_{i} & i=j\\ 0 & i\neq j \end{array}$$

where $N_{i}$ refers to the occupation number of the *i*-th space-orbital. Clearly, for van Vleck states the occupation number is always 1 as all orbitals are singly occupied (no distinction is made for *u* or *d* spin coupling), and being a constant shift for all CSFs^vvs^, it can be omitted when using these terms within the GA fitness function.

The summation over the two-electron excitation operators, $\left\langle m\left|{ê}_{ij,kl}\right|m\right\rangle$ , can be simplified considerably for diagonal Hamiltonian matrix elements, as only terms with {*i*, *j*, *k*, *l*} = {*i*, *i*, *i*, *i*}, {*i*, *i*, *j*, *j*} and {*i*, *j*, *j*, *i*} (*i* ≠ *j*) lead to non-vanishing contributions. As shown in Table IV of ref 18, the $\left\langle m\left|{ê}_{ii,ii}\right|m\right\rangle$ terms are evaluated as *WW*(*d*_*i*_, *d*_*i*_), where *W* refers to *weight operators*, and they vanish for any *d* (or *d*′) ≠ 3. Furthermore, considering that

11
$${ê}_{ii,jj}={Ê}_{ii}{Ê}_{jj}-\delta_{ij}{Ê}_{ij}={Ê}_{ii}{Ê}_{jj},\mathrm{for}\;i\neq j$$

it follows that $\left\langle m\left|{ê}_{ii,jj}\right|m\right\rangle =N_{i}N_{j}$ , and it represents a constant for CSFs composed exclusively of singly occupied orbitals, which can be neglected when designing the GA fitness function. Equation 9 can therefore be expressed as

12
$$\left\langle m\left|Ĥ\right|m\right\rangle =\underset{=const.}{\underset{︸}{\sum_{k,d_{k}\neq 0}h_{kk}N_{k}}}+\underset{=0}{\underset{︸}{\frac{1}{2}\sum_{k,d_{k}=3}g_{kk,kk}WW\left(d_{k},d_{k}\right)}}+\underset{=const.}{\underset{︸}{\frac{1}{2}\sum_{k\neq l}g_{kk,ll}N_{k}N_{l}}}+\frac{1}{2}\sum_{i\neq j}g_{ij,ji}\left\langle m\left|{ê}_{ij,ji}\right|m\right\rangle$$

Since the first three summations either vanish or represent constant shifts within the space of CSFs^vvs^, they can be neglected when using ⟨m|Ĥ|m⟩ as fitness function. The symmetry in ${ê}_{ij,ji}$ and *g*_*ij*,*ji*_ under index permutation further reduces the summation in the last term to *i* < *j*, while removing the 1/2 prefactor

13
$$\left\langle m\left|Ĥ\right|m\right\rangle =\sum_{i<j}g_{ij,ji}\left\langle m\left|{ê}_{ij,ji}\right|m\right\rangle +C$$

where *C* absorbs the constant terms.

Magnetic interactions in various systems are well described by the Heisenberg model Hamiltonian operator (general form)^64–66^

14
$$Ĥ=\sum_{p>q}\left(J_{p,q}^{x}{Ŝ}_{p}^{x}{Ŝ}_{q}^{x}+J_{p,q}^{y}{Ŝ}_{p}^{y}{Ŝ}_{q}^{y}+J_{p,q}^{z}{Ŝ}_{p}^{z}{Ŝ}_{q}^{z}\right)$$

where the indices *p* and *q* are taken over all sites, *J*_*p*,*q*_^*w*^ are exchange coupling constants along the *w* = {*x*, *y*, *z*} directions, and ${Ŝ}_{p}^{w}$ are the quantum spin operators. For systems with isotropic interactions, the exchange coupling parameters are equal for all three directions, i.e., *J*_*p*,*q*_ = *J*_*p*,*q*_^*x*^ = *J*_*p*,*q*_^*y*^ = *J*_*p*,*q*_^*z*^. When only NN interactions are taken into account, the Hamiltonian is further simplified by focusing solely on the exchange coupling parameters *J*_*p*,*q*_^′^, with *p* and *q* being adjacent magnetic sites, indicated by ⟨*p*, *q*⟩ in the summation subscript

15
$$Ĥ=J^{\prime }\sum_{\left\langle p,q\right\rangle }{Ŝ}_{p}\cdot {Ŝ}_{q}=-\frac{J^{\prime }}{2}\sum_{\left\langle p,q\right\rangle }\left({ê}_{pq,qp}+\frac{{ê}_{pp,qq}}{2}\right)$$

The last equation arises from the spin-free formulation of the ${Ŝ}_{p}\cdot {Ŝ}_{q}$ spin–spin interaction. Similarly to the ab initio Hamiltonian operator (eq 12), also for the Heisenberg Hamiltonian operator, the diagonal element $\left\langle m\left|{ê}_{pp,qq}\right|m\right\rangle$ represents a constant shift for all CSFs^vvs^, |*m*⟩, and it can be neglected in the context of GA fitness functions. Moreover, we write the exchange coupling parameter as $J=-\frac{J^{\prime }}{2}$ , leading to

16
$$\left\langle m\left|Ĥ\right|m\right\rangle =J\sum_{\left\langle p,q\right\rangle }\left\langle m\left|{ê}_{pq,qp}\right|m\right\rangle +C^{\prime }$$

where *C*′ accounts for the constant shift. A connection between eqs 16 and 13 is evident.

Following Shavitt’s GUGA method,^18^ the non-constant part of eq 13 (or similarly eq 16) can be further expanded in terms of the *segment values* for the *tail*, *head* and *inner body* segments in the range of the *generators*, (*i*, *j*), namely

17
⟨m|êij,ji|m⟩=[RL_(di,di,x=0)︷=−1/2·∏k=i+1j−1RL(dk,dk,Δb=0,x=0)︷=1·RL®(dj,dj,x=0)︷=1/2]+[RL_(di,di,x=1)·∏k=i+1j−1RL(dk,dk,Δb=0,x=1)·RL®(dj,dj,x=1)︷Tij]

where the expressions for $\underline{RL}\left(d_{i},d_{i}\right)$ , $\prod_{k=i+1}^{j-1}RL\left(d_{k},d_{k},\Delta b=0\right)$ and $\overset{®}{RL}\left(d_{j},d_{j}\right)$ are provided in Table VI of ref 18. As highlighted in eq 17, the first term (*x* = 0) does not depend on the chosen CSF; its effect is to add a constant shift −1/2 (as we are only considering CSFs^vvs^), to the CSF energy, and it can be neglected when using the CSF energy as a fitness function. On the contrary, the second term, *T*_*ij*_, depends on the specific segment values of the |*m*⟩ CSF, and it is responsible for the CSF energy variations. Considering that the segment values for the space of CSFs^vvs^ can only assume the values *d* = 1 and 2, we can rewrite the non-constant part of eq 17 as

18
$$T_{ij}=\{\begin{array}{ll} d=1: & \underline{RL}=\sqrt{\frac{b+2}{2b}},\\ & RL=\sqrt{\frac{\left(b+2\right)\left(b-1\right)}{b\left(b+1\right)}},\\ & \overset{®}{RL}=-\sqrt{\frac{b-1}{2\left(b+1\right)}}\\ d=2: & \underline{RL}=-\sqrt{\frac{b}{2\left(b+2\right)}},\\ & RL=\sqrt{\frac{b\left(b+3\right)}{\left(b+2\right)\left(b+1\right)}},\\ & \overset{®}{RL}=\sqrt{\frac{b+3}{2\left(b+1\right)}} \end{array}$$

where the product of the corresponding segment values is implied. *T*_*ij*_ is equivalent to *X*(*i*, *j*) in ref 45 up to a constant prefactor when only considering the CSFs^vvs^. The two cases (*d* = 1 and *d* = 2) can be combined into a single expression. As only the head or tail segments carry negative signs in eq 18, the sign of the coupling term is determined by the head and tail segment values: ${\left(-1\right)}^{d_{i}+d_{j}+1}$ . Moreover, since the inner body segment values consist of the factors appearing in the head and tail segment values, i.e.

19
$$\begin{array}{l} RL\left(d=1\right)=\sqrt{\frac{\left(b+2\right)\left(b-1\right)}{b\left(b+1\right)}}=\sqrt{\frac{b+2}{b}}\sqrt{\frac{b-1}{b+1}}\\ RL\left(d=2\right)=\sqrt{\frac{b\left(b+3\right)}{\left(b+2\right)\left(b+1\right)}}=\sqrt{\frac{b}{b+2}}\sqrt{\frac{b+3}{b+1}} \end{array}$$

we can restructure the expression as products of head-like and tail-like factors. A generalized form of the head-like factors is obtained by solving for *x* and *c* for numerator and denominator, respectively, in

20
$$\begin{array}{ll} Numerators: & \{\begin{array}{l} b+xd_{1}+c=b-1\\ b+xd_{2}+c=b+3 \end{array}\\ Denominators:, & \{\begin{array}{l} b+xd_{1}+c=b+1\\ b+xd_{2}+c=b+1 \end{array} \end{array}$$

to obtain the expression $\frac{b_{k}+4d_{k}-5}{b_{k}+1}$ that satisfies $\frac{b-1}{b+1}$ for *d* = 1 and $\frac{b+3}{b+1}$ for *d* = 2. A similar generalization is obtained for the tail-like factors, leading to the following general expression for *T*_*ij*_

21
$$T_{ij}=\frac{{\left(-1\right)}^{d_{i}+d_{j}+1}}{2}\sqrt{\prod_{k=i}^{j-1}\frac{b_{k}-2d_{k}+4}{b_{k}+2d_{k}-2}\prod_{k=i+1}^{j}\frac{b_{k}+4d_{k}-5}{b_{k}+1}}$$

and the diagonal matrix element can be expressed as

22
$$\left\langle m\left|Ĥ\right|m\right\rangle =\sum_{i>j}g_{ij,ji}T_{ij}+const.$$

### A.2 Factors Affecting the *T*_*ij*_ Magnitude

Since *T*_*ij*_ depends solely on the selected CSF, increasing the magnitudes of the unmasked *T*_*ij*_ elements can be achieved by selecting a CSF that meets the following criteria:

(i) Increase the number of alternating *ud* couplings.

Starting from eq 5, we define the factors

23
$$\begin{array}{l} P\left(b_{k},d_{k}\right)=\frac{b_{k}-2d_{k}+4}{b_{k}+2d_{k}-2}\\ Q\left(b_{k},d_{k}\right)=\frac{b_{k}+4d_{k}-5}{b_{k}+1} \end{array}$$

making explicit that the magnitude of each *T*_*ij*_ element depends on the products of *P*(*b*_*k*_, *d*_*k*_) and *Q*(*b*_*k*_, *d*_*k*_) over the corresponding range of *k*. Figure 12a shows that *P*(*b*_*k*_, *d*_*k*_) is maximized for *d*_*k*_ = 1 and small *b*_*k*_ (blue), whereas *Q*(*b*_*k*_, *d*_*k*_) is maximized for *d*_*k*_ = 2 and small *b*_*k*_ (orange) as shown in Figure 12b. For the edge indices (*k* = *i* or *k* = *j*), only one factor—either *P*(*b*_*i*_, *d*_*i*_) or *Q*(*b*_*j*_, *d*_*j*_)—contributes to *T*_*ij*_, and for a given *b*_*k*_, its magnitude depends entirely on the corresponding *d*_*k*_. The internal factors (*i* < *k* < *j*) are governed by the product *P*(*b*_*k*_, *d*_*k*_)*Q*(*b*_*k*_, *d*_*k*_). Figure 12c shows that this product is maximized for large *b*_*k*_, asymptotically approaching 1. As it is always less than 1, it reduces the magnitude of *T*_*ij*_, with the reduction becoming more pronounced as *b*_*k*_ decreases.

Considering both edge and internal factors, large *T*_*ij*_ magnitudes are favored when (a) the number of internal *P*(*b*_*k*_, *d*_*k*_)*Q*(*b*_*k*_, *d*_*k*_) factors is minimal or zero, suggesting that |*j* – *i*| should be small (ideally |*j* – *i*| = 1), and (b) the edge factors are large, which occurs when *d*_*i*_ = 1 and *d*_*j*_ = 2 with small *b*_*i*_ and *b*_*j*_. The CSF |*u*_1_ *u*_3_ *d*_2_ *u*_5_ *d*_4_ *d*_6_⟩ of the six-site chain meets these criteria, as its unmasked *T*_*ij*_ elements typically have *d*_*i*_ = 1, *d*_*j*_ = 2, and small |*j* – *i*|. For example, the unmasked contributions associated with site 3 (*i* = 2, *d*_*i*_ = 1) are sites 2 (*j* = 3, *d*_*j*_ = 2) and 4 (*j* = 5, *d*_*j*_ = 2).

(ii) Optimal *b*_*k*_ scales with the coordination number.

Although small *b*_*k*_ favors large |*T*_*ij*_| in the absence of internal factors (*P*(*b*_*k*_, *d*_*k*_)*Q*(*b*_*k*_, *d*_*k*_)), it is not always optimal because the contribution of internal factors is in general unavoidable. As the orbital-index difference |*j* – *i*| increases, more internal factors enter the product, further reducing |*T*_*ij*_|. This reduction is more pronounced for small *b*_*k*_, as shown in Figure 12c. The overall orbital-index difference in a system scales with its coordination number. This can be illustrated by comparing the 2 × 11 lattice in Figure 5 with the 1 × 11 chain. In the natural ordering, site 5 in the 1 × 11 chain is connected to sites 4 and 6, each with an orbital-index difference of 1. In contrast, in the 2 × 11 lattice, site 5 is also connected to site 16, with an orbital-index difference of 16 – 5 = 11. While certain orderings may reduce specific orbital-index differences, they may simultaneously increase others that are smaller in the natural ordering. This implies that in systems with large coordination numbers, and thus larger overall orbital-index differences, the optimal *b*_*k*_ minimizing the CSF energy tends to be larger, due to the stronger influence of internal factors.

Combining these two aspects, we conclude that a CSF yielding a low diagonal energy has the structure

24
$$|\underset{Consecutive\;u}{\underset{︸}{u...u}}\overset{Internal\;alternating\;ud}{\overset{︷}{ud...ud}}\underset{Consecutive\;d}{\underset{︸}{d...d}}\rangle$$

where the number of consecutive *u* couplings at the beginning (and *d* at the end) increases with the coordination number or lattice dimensionality. The remaining couplings alternate between *u* and *d* in the internal section. Accordingly, more consecutive initial *u* couplings (i.e., larger *b*_*k*_) are expected in 3D Heisenberg models than in 2D or 1D, *b*_*k*_^3D^ > *b*_*k*_^2D^ > *b*_*k*_^1D^. This trend is demonstrated numerically in Section 4.

## Notes

(A) Computational details of the calculations reported in this work and representative input files for all calculations reported in this work, (B) CSF energy plots of the 8-site model systems not presented in the main text, (C) a short summary of the Schur–Horn theorem, (D) Hamiltonian matrices for the 6-site Heisenberg chain, (E) GA results for the 4 × 4, 5 × 5, and 6 × 6 square lattices, and (F) FCIQMC energies of the 14 collinear singlet states for the P-cluster (PDF)
A ZIP archive is also provided, which contains the input files listed in (A) along with the corresponding FCIDUMP files for the 2 × 11 Heisenberg model and the CAS­(48,40) active space for the P-cluster (ZIP)
