# Supplementary material for: A Genetic Algorithm Approach for Compact Wave Function Representations in Spin-Adapted Bases
Source: J Chem Theory Comput. 2025 Nov 10;21(22):11533–49. doi: 10.1021/acs.jctc.5c01264 (PMC12659022; doi:10.1021/acs.jctc.5c01264)
Supplement: Supplementary file 1 [file ct5c01264_si_001.pdf]

Supporting Material: A Genetic Algorithm Approach for Compact Wave Function Representations in Spin-Adapted Bases

Maru Song and Giovanni Li Manni*

Max Planck Institute for Solid State Research, 70569 Stuttgart, Germany

E-mail: g.limanni@fkf.mpg.de

Supporting Information Available

S1 Computational Details

Active Space Preparation. The geometry of the resting state of the P-cluster (P^N) was taken from Reference 1. To create the simple CAS(8,8) H_8 cluster model, we replaced the Fe atoms with H atoms and removed all other atoms. Unrestricted Hartree-Fock (UHF) orbitals were generated and localized using the Pipek-Mezey localization method.[2] A minimal basis set was adopted, using relativistic atomic natural orbitals (ANO-RCC-MB).

For the more realistic P-cluster in its P^N resting state, a larger basis set was employed, where the Fe atoms were described by the ANO-RCC-VDZP basis, the S atoms by the ANO-RCC-VDZ basis, and the remaining atoms by the ANO-RCC-MB basis. The UHF procedure was followed by the restricted open-shell Hartree-Fock (ROHF) calculation. The ROHF doubly and singly occupied orbitals were localized separately using the Pipek–Mezey method,[2] to preserve orbital invariance and the character of the doubly occupied orbitals.

The localized 3d orbitals of the 8 Fe(II) atoms and their electrons form the CAS(48,40) active space. In the CAS(114,73) active space, the CAS(48,40) was expanded by including three 3p orbitals from the 7 sulfur bridging atoms and two 3p orbitals from the 6 peripheral S atoms. The active space molecular orbital integrals were written in the `FCIDUMP` format[3] using routines written by G. Li Manni in the `OpenMolcas` codebase.[4] All calculations for this active space preparation step were carried out using `OpenMolcas`.[4] The evaluation of the electron repulsion integrals has been greatly simplified by means of the resolution-of-identity Cholesky-decomposition technique as implemented in the `OpenMolcas` software,[4–6] with a decomposition threshold of 10^{-4} a.u.[7–11] Scalar relativistic effects were introduced via second order Douglas–Kroll–Hess integral correction.

FCIQMC. Spin-adapted FCIQMC[12,13] calculations were performed using the `NECI` codebase.[14] For *ab initio* systems, we used the excited-state FCIQMC technique,[15] that samples the ground and excited states by simultaneously running multiple FCIQMC dynamics that are orthogonal to each other. We also used the initiator approximation[16–18] and spawn truncation with the semi-stochastic adaptation[19,20] to perform more efficient calculations. The Pre-Computed Heat Bath (PCHB) excitation generator has been employed, which suggests excitations with large couplings more often.[14,21–24]

Details on GA. An *ex novo* Python-based GA code was developed to optimize the orbital ordering of the GUGA basis, and is available as part of the `OpenMolcas` auxiliary tools.[4] In input, the code requires the FCIDUMP file (or information on the J magnetic couplings) for the active space under consideration (only magnetic orbitals), and a file with the GA-related input parameters, including population size, elite size, number of generations, mutation, crossover, cluster reshuffling, and distribution of orbitals across the magnetic sites. In output, the code produces a file containing information about the GA progress, including the best fitness value per generation and the corresponding site ordering, a file containing information about the latest GA population, and a new FCIDUMP file, which contains the electron

repulsion integrals labeled according to the optimal orbital ordering.

DMRG. For benchmarking purposes, we used `BLOCK`[25–28] to obtain spin-adapted DMRG benchmark energy of the NN Heisenberg model on the 2×11 lattice.

S1.1 Input Files

S1.1.1 `NECI` input for the 2×11 Heisenberg lattice.

```
# NECI input for 2x11 NN Heisenberg
Title

System read
    electrons 22
    nonuniformrandexcits guga-pchb localised
    nobrillouintheorem
    guga 0
    freeformat
    FCIDUMP-name FCIDUMP_2x11_GA
endsys

calc
    seed -1

    methods
        method vertex fcimc
    endmethods

    definedet 1 3 6 7 10 11 14 15 18 19 22 23 26 27 30 31 34 35 38 39
    42 44

    totalwalkers     5.e4
    tau-values \
        ignore-diagonal-estimate \
        start user-defined 0.01

    target-shiftDamp 0.5
    diagshift -10
    stepsshift 10
    nmcyc 800000

    proje-changeref 1.2

    allrealcoeff
    realspawncutoff 0.03
    jump-shift
```

```
    memoryfacspawn 100
    memoryfacpart 50

    time 1400

    startsinglepart 100
endcalc

logging
    highlypopwrite 2000
    hdf5-pops
endlog

end
```

S1.1.2 BLOCK input for the 2×11 Heisenberg lattice.

```
nelec 22
spin 0
irrep 1

hf_occ integral
schedule default
maxM 1000
maxiter 30

prefix /scratch/song/block/

orbitals FCIDUMP_2x11_GA
```

S1.1.3 OpenMolcas input files for CAS(114,73) orbitals.

UHF

```
&GATEWAY
    RICD
    CDThreshold = 1.0d-4
    Coord = PN-cluster.xyz
    basis = FE.ANO-RCC-VDZP, S.ANO-RCC-VDZ, ANO-RCC-MB
    group = nosym

&SEWARD

&SCF
    UHF
```

```
    zspin = 32
    charge = -4
    PrOrb = 2 1.0d10 3
    Thresholds = 5.0d-8 5.0d-4 1.0d-4 5.0d-3

>>> COPY $CurrDir/$Project.UnaOrb INPORB
```

ROHF

```
&GATEWAY
    RICD
    CDThreshold = 1.0d-4
    Coord = PN-cluster.xyz
    basis = FE.ANO-RCC-VDZP, S.ANO-RCC-VDZ, ANO-RCC-MB
    group = nosym

&SEWARD

>>> COPY $CurrDir/$Project.UnaOrb INPORB
&RASSCF
    LumOrb
    nactel = 32
    spin = 33
    inactive = 350
    ras2 = 32
```

Split localization

```
&GATEWAY
    RICD
    CDThreshold = 1.0d-4
    Coord = PN-cluster.xyz
    basis = FE.ANO-RCC-VDZP, S.ANO-RCC-VDZ, ANO-RCC-MB
    group = nosym

&SEWARD

&LOCALISATION
      FILEorb = PN.RasOrb.1
      PIPE
      NFRO = 145
      NORB = 205

&LOCALISATION
      FILEorb = PN.LocOrb
      PIPE
      NFRO = 350
      NORB = 32
```

S1.1.4 NECI input for the CAS(48,40) P-cluster

```
# NECI input for CAS(48,40) P-cluster
# This input uses the excitated state feature,
# Thus it should be run with `mneci` executable.

Title

System read
    electrons 48
    nonuniformrandexcits guga-pchb localised
    nobrillouintheorem
    guga 0
    freeformat
    system-replicas 14
endsys

calc
    multiple-initial-states
    1 2 3 5 7 9   11 12 14 16 18 20   21 22 23 25 27 29   31 32 34 36
    38 40   41 42 43 45 47 49   51 52 54 56 58 60   61 62 63 65 67 69
      71 72 74 76 78 80
    1 2 3 5 7 9   11 12 14 16 18 20   21 22 23 25 27 29   31 32 34 36
    38 40   41 42 43 45 47 49   51 52 53 55 57 59   61 62 64 66 68 70
      71 72 74 76 78 80
    1 2 3 5 7 9   11 12 13 15 17 19   21 22 24 26 28 30   31 32 34 36
    38 40   41 42 43 45 47 49   51 52 54 56 58 60   61 62 63 65 67 69
      71 72 74 76 78 80
    1 2 3 5 7 9   11 12 13 15 17 19   21 22 24 26 28 30   31 32 34 36
    38 40   41 42 43 45 47 49   51 52 53 55 57 59   61 62 64 66 68 70
      71 72 74 76 78 80
    1 2 3 5 7 9   11 12 13 15 17 19   21 22 23 25 27 29   31 32 34 36
    38 40   41 42 44 46 48 50   51 52 53 55 57 59   61 62 64 66 68 70
      71 72 74 76 78 80
    1 2 3 5 7 9   11 12 14 16 18 20   21 22 23 25 27 29   31 32 33 35
    37 39   41 42 43 45 47 49   51 52 54 56 58 60   61 62 64 66 68 70
      71 72 74 76 78 80
    1 2 3 5 7 9   11 12 13 15 17 19   21 22 23 25 27 29   31 32 34 36
    38 40   41 42 44 46 48 50   51 52 54 56 58 60   61 62 63 65 67 69
      71 72 74 76 78 80
    1 2 3 5 7 9   11 12 13 15 17 19   21 22 23 25 27 29   31 32 34 36
    38 40   41 42 43 45 47 49   51 52 54 56 58 60   61 62 64 66 68 70
      71 72 74 76 78 80
    1 2 3 5 7 9   11 12 14 16 18 20   21 22 23 25 27 29   31 32 33 35
    37 39   41 42 44 46 48 50   51 52 54 56 58 60   61 62 63 65 67 69
      71 72 74 76 78 80
    1 2 3 5 7 9   11 12 13 15 17 19   21 22 24 26 28 30   31 32 33 35
    37 39   41 42 44 46 48 50   51 52 53 55 57 59   61 62 64 66 68 70
      71 72 74 76 78 80
```

```
1 2 3 5 7 9   11 12 14 16 18 20   21 22 23 25 27 29   31 32 33 35
37 39   41 42 44 46 48 50   51 52 53 55 57 59   61 62 64 66 68 70
  71 72 74 76 78 80
1 2 3 5 7 9   11 12 13 15 17 19   21 22 24 26 28 30   31 32 33 35
37 39   41 42 44 46 48 50   51 52 54 56 58 60   61 62 63 65 67 69
  71 72 74 76 78 80
1 2 3 5 7 9   11 12 13 15 17 19   21 22 24 26 28 30   31 32 33 35
37 39   41 42 43 45 47 49   51 52 54 56 58 60   61 62 64 66 68 70
  71 72 74 76 78 80
1 2 3 5 7 9   11 12 13 15 17 19   21 22 23 25 27 29   31 32 33 35
37 39   41 42 44 46 48 50   51 52 54 56 58 60   61 62 64 66 68 70
  71 72 74 76 78 80

seed -1

methods
    method vertex fcimc
endmethods

totalwalkers     1.e6
tau-values \
    ignore-diagonal-estimate \
    start user-defined 0.001

semi-stochastic 3000
pops-core 100000

diagshift 0.00
#shiftdamp 0.02
target-shiftDamp 0.5
stepsshift 10

nmcyc 100000

proje-changeref 1.5
no-changeref

truncinitiator
addtoinitiator 10

truncate-spawns 30

allrealcoeff
realspawncutoff 0.03

memoryfacspawn 50
memoryfacpart 30

time 2800
```

```
    startsinglepart 100

    orthogonalise-replicas-symmetric
    replica-single-det-start
endcalc

logging
    highlypopwrite 2000
    hdf5-pops
endlog

end
```

S2 CSF Energies Over Different Orderings

Figure S1 shows the lowest and highest diagonal matrix elements, and their difference (ΔE_{diag}) for cases (c), (d), (e), and (f).

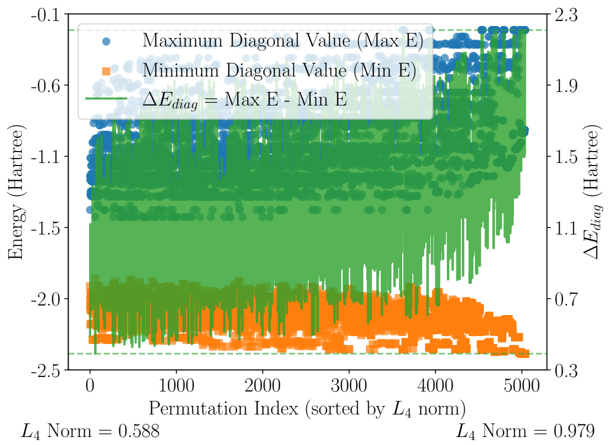

(c) Heisenberg P-cluster ($J_{ij} \propto 1/r_{ij}$).

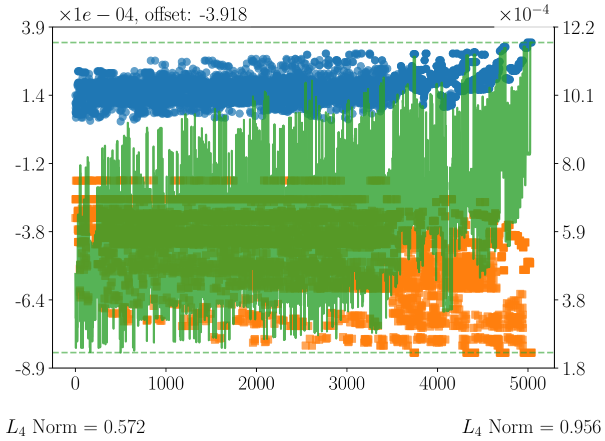

(d) H_8 model of the P-cluster.

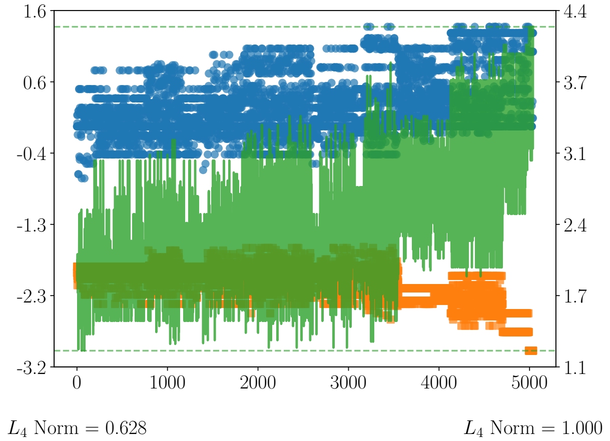

(e) 8-site $J_2/J_1 = 0.5$ Heisenberg chain.

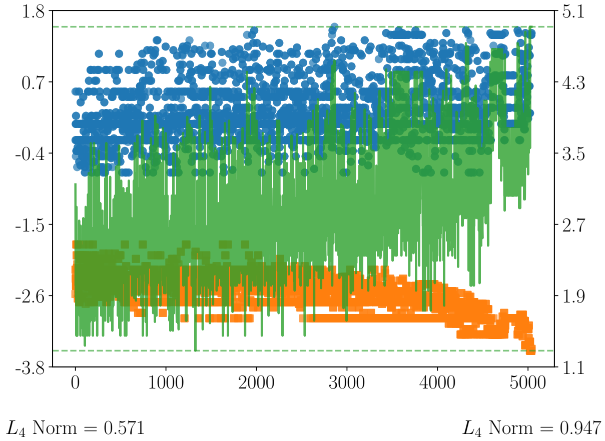

(f) 8-site $J_2/J_1 = 1.0$ Heisenberg chain.

Figure S1: Lowest (yellow squares) and highest (blue circles) diagonal matrix elements, $\langle m|\hat{\mathcal{H}}|m\rangle$, over the van Vleck-Sherman configuration space, and their difference (ΔE_{diag}, green line) for four different test cases. The left y-axis shows the value of the diagonal matrix elements, while the right y-axis shows the ΔE_{diag} value.

S3 The Schur-Horn Theorem

The Schur-Horn theorem[29] builds a connection between the eigenvalues, $\lambda(B) = [\lambda_i(B)]$ (in non-increasing order), and the diagonal elements, $a(B) = [a_{ii}(B)]$ (in non-increasing order),

of a Hermitian matrix, $B^{n\times n}$, stating that $\lambda(B)$ *majorizes* $a(B)$ ($\lambda(B) \succ a(B)$):

$$\sum_{i=1}^{k} \lambda_i(B) \geq \sum_{i=1}^{k} a_{ii}(B) \quad \forall\, k = 1, 2, \ldots, n-1\,, \tag{1}$$

$$\sum_{i=1}^{k} \lambda_i(B) = \sum_{i=1}^{k} a_{ii}(B) \quad \text{for} \quad k = n\,, \tag{2}$$

where in Eq. 2 the equality for $k = n$ arises from the fact that both sides of the equation sum to $\mathrm{tr}(B)$. For $k = 1$, the relation trivially implies that the largest eigenvalue, $\lambda_1(B)$, is greater than or equal to the largest diagonal element $a_{11}(B)$:

$$\lambda_1(B) \geq a_{nn}(B)\,. \tag{3}$$

Subtracting from Eq. 2 the sum of the first $n-1$ elements (Eq. 1) leads to

$$\lambda_n(B) \leq a_{nn}(B)\,, \tag{4}$$

that is, the smallest eigenvalue is smaller than or equal to the smallest diagonal entry. Therefore, maximizing the gap between the largest and smallest diagonal elements by application of site permutations approaches the largest and smallest eigenvalues, which is the theoretical limit for a transformation that diagonalizes the Hamiltonian matrix and maximizes sparsity.

S4 Six-site NN Heisenberg Hamiltonian

Figure S2 shows the Hamiltonian matrices for the Six-site NN Heisenberg chain.

S5 4×4, 5×5, and 6×6 Square Lattice Results

We applied the GA ordering optimization to 4×4, 5×5, and 6×6 square lattices. For these cases, we used 200, 1000, and 5000 chromosomes per generation, respectively. Figure S3

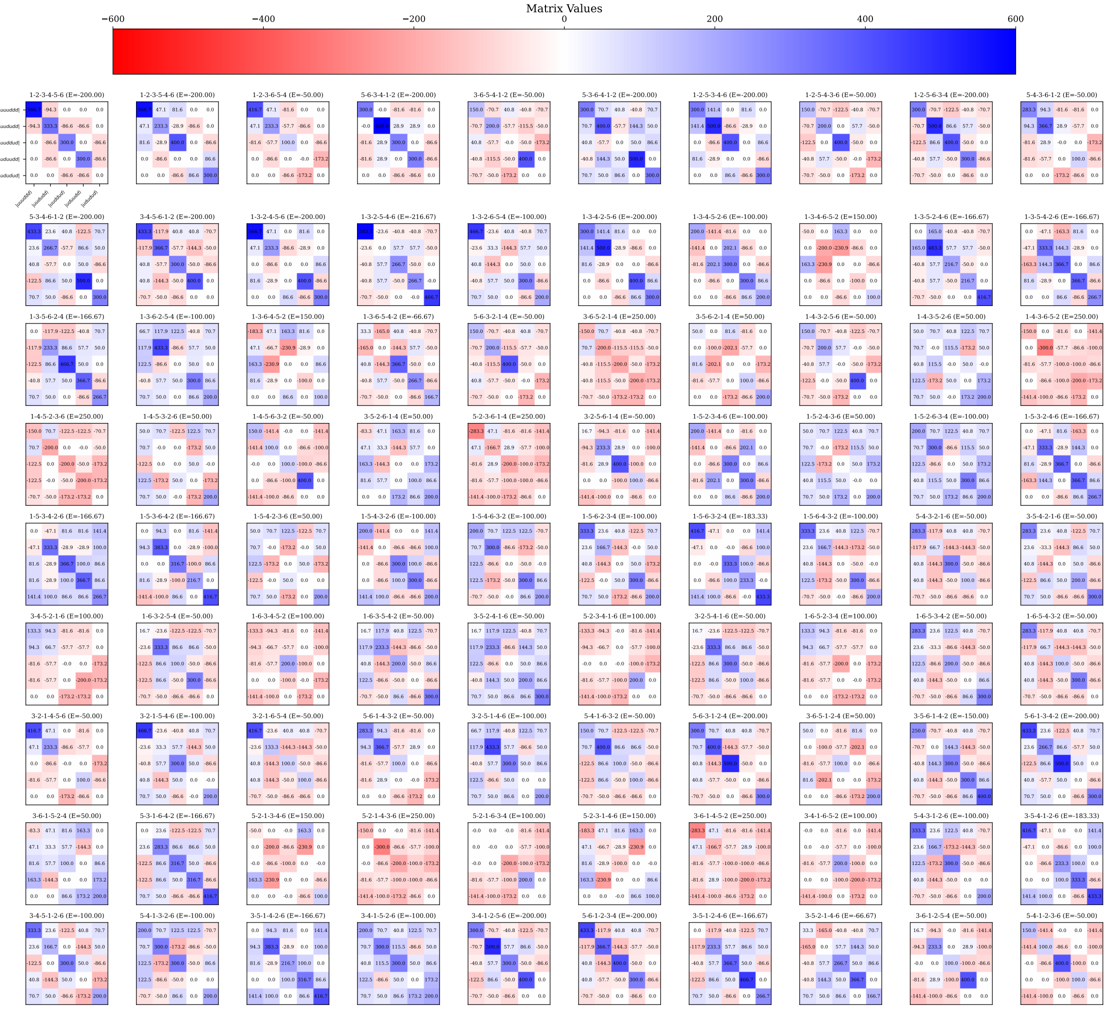

Figure S2: Hamiltonian matrices for the Six-site NN Heisenberg chain with open boundaries ($J = -100$) only for the symmetry non-equivalent permutations.

displays the resulting GA orderings and the corresponding S–M_S-consistent CSFs. As the lattice size increases, the overall connectivity grows (due to a decreasing perimeter-to-area ratio), leading to a larger number of initial u couplings in the optimal ordering.

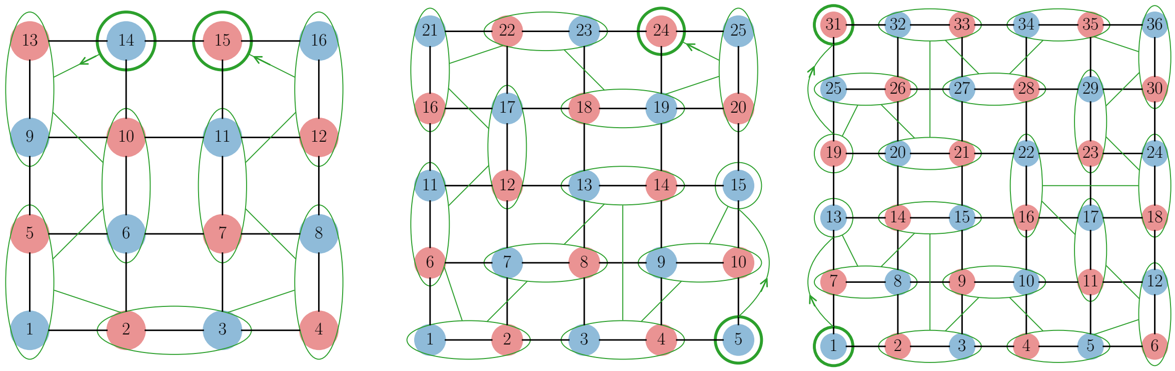

(a) 4×4. (b) 5×5. (c) 6×6.

Figure S3: Square lattices with the corresponding GA-found optimal orderings and the S–M_S-consistent CSFs. Blue and red circles denote u and d spin couplings, respectively, with site labels indicated. Green lines trace the GA-optimized ordering, starting at the first u coupling and ending at the last d coupling. Thick green circles mark the start and end of the sequence, while alternating ud pairs are highlighted by green ellipses.

S6 P-cluster FCIQMC Results

Table S1 shows the energies of the 14 collinear singlet states for CAS(48,40) and CAS(114,73) of the P-clsuter.

Table S1: Comparison of electronic state energies between CAS(48,40) and CAS(114,73) calculations using the Fe_8-GA ordering, $(3-4-2-1-8-7-5-6)$. Weights of the leading CSF in each state are taken from the final iteration of the corresponding FCIQMC wave functions

	CAS(48,40)		CAS(114,73)	
State	Energy[a] (mHa)	Weight (%)	Energy[a] (mHa)	Weight (%)
$\|UUUDUDDD\rangle$	-513.673 ± 0.002	97.2	-525.240 ± 0.012	94.7
$\|UDUUUDDD\rangle$	-507.418 ± 0.009	96.1	-517.520 ± 0.015	91.0
$\|UUDUUDDD\rangle$	-505.734 ± 0.004	97.5	-516.212 ± 0.036	94.6
$\|UUUDDUDD\rangle$	-504.942 ± 0.003	97.8	-515.381 ± 0.007	95.0
$\|UUDDUUDD\rangle$	-505.040 ± 0.007	96.5	-514.142 ± 0.011	90.8
$\|UUUDDDUD\rangle$	-504.307 ± 0.012	97.1	-513.376 ± 0.018	93.2
$\|UUDDUDUD\rangle$	-505.625 ± 0.029	90.0	-512.749 ± 0.021	84.8
$\|UDUDUUDD\rangle$	-503.358 ± 0.025	94.0	-511.353 ± 0.031	85.5
$\|UDUDUDUD\rangle$	-503.777 ± 0.040	77.3	-510.071 ± 0.029	76.2
$\|UDUUDUDD\rangle$	-498.752 ± 0.017	96.3	-507.929 ± 0.025	89.2
$\|UDUUDDUD\rangle$	-498.431 ± 0.013	93.7	-506.144 ± 0.037	84.2
$\|UUDUDUDD\rangle$	-497.178 ± 0.004	98.0	-506.722 ± 0.019	94.4
$\|UUDUDDUD\rangle$	-496.479 ± 0.007	96.8	-504.985 ± 0.011	91.4
$\|UUUUDDDD\rangle$	-494.090 ± 0.000	99.7	-503.354 ± 0.004	98.7
Lowest-to-Highest Gap	19.583 ± 0.002		21.886 ± 0.013	

[a] Shifted by +17358 Ha.

References

(1) Li, Z.; Guo, S.; Sun, Q.; Chan, G. K.-L. Electronic landscape of the P-cluster of nitrogenase as revealed through many-electron quantum wavefunction simulations. *Nature Chemistry* **2019**, *11*, 1026–1033.

(2) Pipek, J.; Mezey, P. G. A fast intrinsic localization procedure applicable for ab initio and semiempirical linear combination of atomic orbital wave functions. *J. Chem. Phys.* **1989**, *90*, 4916–4926.

(3) Knowles, P. J.; Handy, N. C. Unlimited full configuration interaction calculations. *J. Chem. Phys.* **1989**, *91*, 2396–2398.

(4) Li Manni, G.; Galván, I. F.; Alavi, A.; Aleotti, F.; Aquilante, F.; Autschbach, J.;

Avagliano, D.; Baiardi, A.; Bao, J. J.; Battaglia, S.; Birnoschi, L.; Blanco-González, A.; Bokarev, S. I.; Broer, R.; Cacciari, R.; Calio, P. B.; Carlson, R. K.; Couto, R. C.; Cerdán, L.; Chibotaru, L. F.; Chilton, N. F.; Church, J. R.; Conti, I.; Coriani, S.; Cuéllar-Zuquin, J.; Daoud, R. E.; Dattani, N.; Decleva, P.; de Graaf, C.; Delcey, M. G.; Vico, L. D.; Dobrautz, W.; Dong, S. S.; Feng, R.; Ferré, N.; Filatov(Gulak), M.; Gagliardi, L.; Garavelli, M.; González, L.; Guan, Y.; Guo, M.; Hennefarth, M. R.; Hermes, M. R.; Hoyer, C. E.; Huix-Rotllant, M.; Jaiswal, V. K.; Kaiser, A.; Kaliakin, D. S.; Khamesian, M.; King, D. S.; Kochetov, V.; Krośnicki, M.; Kumaar, A. A.; Larsson, E. D.; Lehtola, S.; Lepetit, M.-B.; Lischka, H.; Ríos, P. L.; Lundberg, M.; Ma, D.; Mai, S.; Marquetand, P.; Merritt, I. C. D.; Montorsi, F.; Mörchen, M.; Nenov, A.; Nguyen, V. H. A.; Nishimoto, Y.; Oakley, M. S.; Olivucci, M.; Oppel, M.; Padula, D.; Pandharkar, R.; Phung, Q. M.; Plasser, F.; Raggi, G.; Rebolini, E.; Reiher, M.; Rivalta, I.; Roca-Sanjuán, D.; Romig, T.; Safari, A. A.; Sánchez-Mansilla, A.; Sand, A. M.; Schapiro, I.; Scott, T. R.; Segarra-Martí, J.; Segatta, F.; Sergentu, D.-C.; Sharma, P.; Shepard, R.; Shu, Y.; Staab, J. K.; Straatsma, T. P.; Sørensen, L. K.; Tenorio, B. N. C.; Truhlar, D. G.; Ungur, L.; Vacher, M.; Veryazov, V.; Voß, T. A.; Weser, O.; Wu, D.; Yang, X.; Yarkony, D.; Zhou, C.; Zobel, J. P.; Lindh, R. The OpenMolcas Web: A Community-Driven Approach to Advancing Computational Chemistry. *Journal of Chemical Theory and Computation* **2023**, 6933–6991.

(5) Aquilante, F.; Autschbach, J.; Carlson, R. K.; Chibotaru, L. F.; Delcey, M. G.; De Vico, L.; Fdez. Galván, I.; Ferré, N.; Frutos, L. M.; Gagliardi, L.; Garavelli, M.; Giussani, A.; Hoyer, C. E.; Li Manni, G.; Lischka, H.; Ma, D.; Malmqvist, P.-Å.; Müller, T.; Nenov, A.; Olivucci, M.; Pedersen, T. B.; Peng, D.; Plasser, F.; Pritchard, B.; Reiher, M.; Rivalta, I.; Schapiro, I.; Segarra-Martí, J.; Stenrup, M.; Truhlar, D. G.; Ungur, L.; Valentini, A.; Vancoillie, S.; Veryazov, V.; Vysotskiy, V. P.; Weingart, O.; Zapata, F.; Lindh, R. Molcas 8: New Capabilities for Multiconfigurational Quantum Chemical Calculations Across the Periodic Table. *J. Comput. Chem.* **2016**, *37*, 506–541.

(6) Fdez. Galván, I.; Vacher, M.; Alavi, A.; Angeli, C.; Aquilante, F.; Autschbach, J.; Bao, J. J.; Bokarev, S. I.; Bogdanov, N. A.; Carlson, R. K.; Chibotaru, L. F.; Creutzberg, J.; Dattani, N.; Delcey, M. G.; Dong, S. S.; Dreuw, A.; Freitag, L.; Frutos, L. M.; Gagliardi, L.; Gendron, F.; Giussani, A.; González, L.; Grell, G.; Guo, M.; Hoyer, C. E.; Johansson, M.; Keller, S.; Knecht, S.; Kovačević, G.; Källman, E.; Li Manni, G.; Lundberg, M.; Ma, Y.; Mai, S.; Malhado, J. a. P.; Malmqvist, P. A.; Marquetand, P.; Mewes, S. A.; Norell, J.; Olivucci, M.; Oppel, M.; Phung, Q. M.; Pierloot, K.; Plasser, F.; Reiher, M.; Sand, A. M.; Schapiro, I.; Sharma, P.; Stein, C. J.; Sørensen, L. K.; Truhlar, D. G.; Ugandi, M.; Ungur, L.; Valentini, A.; Vancoillie, S.; Veryazov, V.; Weser, O.; Wesołowski, T. A.; Widmark, P.-O.; Wouters, S.; Zech, A.; Zobel, J. P.; Lindh, R. OpenMolcas: From Source Code to Insight. *J. Chem. Theory Comput.* **2019**, *15*, 5925–5964.

(7) Aquilante, F.; Pedersen, T. B.; Lindh, R. Low-Cost Evaluation of the Exchange Fock Matrix from Cholesky and Density Fitting Representations of the Electron Repulsion Integrals. *J. Chem. Phys.* **2007**, *126*, 194106.

(8) Aquilante, F.; Lindh, R.; Pedersen, T. B. Unbiased Auxiliary Basis Sets for Accurate Two-Electron Integral Approximations. *J. Chem. Phys.* **2007**, *127*, 114107.

(9) Aquilante, F.; Pedersen, T. B.; Lindh, R.; Roos, B. O.; de Merás, A. S.; Koch, H. Accurate Ab Initio Density Fitting for Multiconfigurational Self-Consistent Field Methods. *J. Chem. Phys.* **2008**, *129*, 024113.

(10) Aquilante, F.; Gagliardi, L.; Pedersen, T. B.; Lindh, R. Atomic Cholesky Decompositions: A Route to Unbiased Auxiliary Basis Sets for Density Fitting Approximation with Tunable Accuracy and Efficiency. *J. Chem. Phys.* **2009**, *130*, 154107.

(11) Pedersen, T. B.; Aquilante, F.; Lindh, R. Density Fitting with Auxiliary Basis Sets from Cholesky Decompositions. *Theor. Chem. Acc.* **2009**, *124*, 1–10.

(12) Dobrautz, W.; Smart, S. D.; Alavi, A. Efficient formulation of full configuration interaction quantum Monte Carlo in a spin eigenbasis via the graphical unitary group approach. *J. Chem. Phys.* **2019**, *151*, 094104.

(13) Dobrautz, W. Development of Full Configuration Interaction Quantum Monte Carlo Methods for Strongly Correlated Electron Systems. Ph.D. thesis, University of Stuttgart, 2019.

(14) Guther, K.; Anderson, R. J.; Blunt, N. S.; Bogdanov, N. A.; Cleland, D.; Dattani, N.; Dobrautz, W.; Ghanem, K.; Jeszenszki, P.; Liebermann, N.; Li Manni, G.; Lozovoi, A. Y.; Luo, H.; Ma, D.; Merz, F.; Overy, C.; Rampp, M.; Samanta, P. K.; Schwarz, L. R.; Shepherd, J. J.; Smart, S. D.; Vitale, E.; Weser, O.; Booth, G. H.; Alavi, A. NECI: N-Electron Configuration Interaction with an emphasis on state-of-the-art stochastic methods. *The Journal of Chemical Physics* **2020**, *153*, 034107.

(15) Blunt, N. S.; Smart, S. D.; Booth, G. H.; Alavi, A. An excited-state approach within full configuration interaction quantum Monte Carlo. *J. Chem. Phys.* **2015**, *143*, 134117.

(16) Cleland, D.; Booth, G. H.; Alavi, A. Communications: Survival of the fittest: Accelerating convergence in full configuration-interaction quantum Monte Carlo. *J. Chem. Phys.* **2010**, *132*, 041103.

(17) Cleland, D.; Booth, G. H.; Alavi, A. A Study of Electron Affinites Using the Initiator Approach to Full Configuration Interaction Quantum Monte Carlo. *J. Chem. Phys.* **2011**, *134*, 024112.

(18) Cleland, D. M. the Initiator Full Configuration Interaction Quantum Monte Carlo Method: Development and Applications to Molecular Systems. Ph.D. thesis, University of Cambridge, 2012.

(19) Petruzielo, F. R.; Holmes, A. A.; Changlani, H. J.; Nightingale, M. P.; Umrigar, C. J. Semistochastic Projector Monte Carlo Method. *Phys. Rev. Lett.* **2012**, *109*, 230201.

(20) Blunt, N. S.; Smart, S. D.; Kersten, J. A. F.; Spencer, J. S.; Booth, G. H.; Alavi, A. Semi-stochastic full configuration interaction quantum Monte Carlo: Developments and application. *J. Chem. Phys.* **2015**, *142*, 184107.

(21) Holmes, A. A.; Changlani, H. J.; Umrigar, C. J. Efficient Heat-Bath Sampling in Fock Space. *J. Chem. Theory Comput.* **2016**, *12*, 1561–1571.

(22) Holmes, A. A.; Tubman, N. M.; Umrigar, C. J. Heat-Bath Configuration Interaction: An Efficient Selected Configuration Interaction Algorithm Inspired by Heat-Bath Sampling. *J. Chem. Theory Comput.* **2016**, *12*, 3674–3680.

(23) Neufeld, V. A.; Thom, A. J. W. Exciting Determinants in Quantum Monte Carlo: Loading the Dice with Fast, Low-Memory Weights. *Journal of Chemical Theory and Computation* **2019**, *15*, 127–140.

(24) Weser, O.; Dobrautz, W.; Li Manni, G. (Unpublished work).

(25) Chan, G. K.-L.; Head-Gordon, M. Highly correlated calculations with a polynomial cost algorithm: A study of the density matrix renormalization group. *J. Chem. Phys.* **2002**, *116*, 4462–4476.

(26) Chan, G. K.-L. An algorithm for large scale density matrix renormalization group calculations. *The Journal of Chemical Physics* **2004**, *120*, 3172–3178.

(27) Ghosh, D.; Hachmann, J.; Yanai, T.; Chan, G. K.-L. Orbital optimization in the density matrix renormalization group, with applications to polyenes and β-carotene. *J. Chem. Phys.* **2008**, *128*, 144117.

(28) Sharma, S.; Chan, G. K.-L. Spin-adapted density matrix renormalization group algorithms for quantum chemistry. *The Journal of Chemical Physics* **2012**, *136*, 124121.

(29) Horn, R. A.; Johnson, C. R. *Matrix Analysis*, second edition, corrected reprint ed.; Cambridge University Press: New York, NY, 2017; Chapter 4.
